# Leveraging genetic diversity in mice to inform individual differences in brain microstructure and memory

**DOI:** 10.3389/fnbeh.2022.1033975

**Published:** 2023-01-10

**Authors:** Thomas J. Murdy, Amy R. Dunn, Surjeet Singh, Maria A. Telpoukhovskaia, Shanrong Zhang, Jacqueline K. White, Itamar Kahn, Marcelo Febo, Catherine C. Kaczorowski

**Affiliations:** ^1^The Jackson Laboratory, Bar Harbor, ME, United States; ^2^Department of Neuroscience, Zuckerman Mind Brain Behavior Institute, Columbia University, New York, NY, United States; ^3^Department of Neuroscience, University of Florida College of Medicine, Gainesville, FL, United States

**Keywords:** Alzheimer’s disease, brain reserve, genetic diversity, 5XFAD, memory, NODDI, DTI, diffusion MRI (dMRI)

## Abstract

In human Alzheimer’s disease (AD) patients and AD mouse models, both differential pre-disease brain features and differential disease-associated memory decline are observed, suggesting that certain neurological features may protect against AD-related cognitive decline. The combination of these features is known as brain reserve, and understanding the genetic underpinnings of brain reserve may advance AD treatment in genetically diverse human populations. One potential source of brain reserve is brain microstructure, which is genetically influenced and can be measured with diffusion MRI (dMRI). To investigate variation of dMRI metrics in pre-disease-onset, genetically diverse AD mouse models, we utilized a population of genetically distinct AD mice produced by crossing the 5XFAD transgenic mouse model of AD to 3 inbred strains (C57BL/6J, DBA/2J, FVB/NJ) and two wild-derived strains (CAST/EiJ, WSB/EiJ). At 3 months of age, these mice underwent diffusion magnetic resonance imaging (dMRI) to probe neural microanatomy in 83 regions of interest (ROIs). At 5 months of age, these mice underwent contextual fear conditioning (CFC). Strain had a significant effect on dMRI measures in most ROIs tested, while far fewer effects of sex, sex*strain interactions, or strain*sex*5XFAD genotype interactions were observed. A main effect of 5XFAD genotype was observed in only 1 ROI, suggesting that the 5XFAD transgene does not strongly disrupt neural development or microstructure of mice in early adulthood. Strain also explained the most variance in mouse baseline motor activity and long-term fear memory. Additionally, significant effects of sex and strain*sex interaction were observed on baseline motor activity, and significant strain*sex and sex*5XFAD genotype interactions were observed on long-term memory. We are the first to study the genetic influences of brain microanatomy in genetically diverse AD mice. Thus, we demonstrated that strain is the primary factor influencing brain microstructure in young adult AD mice and that neural development and early adult microstructure are not strongly altered by the 5XFAD transgene. We also demonstrated that strain, sex, and 5XFAD genotype interact to influence memory in genetically diverse adult mice. Our results support the usefulness of the 5XFAD mouse model and convey strong relationships between natural genetic variation, brain microstructure, and memory.

## 1. Introduction

Alzheimer’s disease (AD) is a fatal neurodegenerative disease that is the leading cause of dementia worldwide (Alzheimer’s Association, 2021) and is currently untreatable. It has been noted that some individuals require larger levels of neurodegeneration or molecular pathology to develop cognitive symptoms of AD and other neurodegenerative diseases (Satz, 1993; Koepsell et al., 2008; Stern, 2012). These observations gave rise to the theory of brain reserve, which states that certain brain features may maintain cognitive health in individuals with AD-related neurological pathology. By identifying sources of brain reserve and their genetic underpinnings, new biomarkers and therapeutic targets for AD can be developed. One potential source of brain reserve is the microstructural organization of the brain, such as axon and dendrite architecture. These anatomical features can be revealed with high accuracy using diffusion magnetic resonance imaging (dMRI).

Diffusion magnetic resonance imaging is a magnetic resonance imaging (MRI) technique that can characterize brain microstructure by determining the restriction of water molecule displacement during diffusion in neural tissue. Different information can be obtained from dMRI signals, depending on the mathematical model used to process raw dMRI data. Two models commonly used in neuroimaging are diffusion tensor imaging (DTI), which conveys general information about brain tissue composition based on Gaussian models of water diffusion (Basser et al., 1994a,b), and neurite orientation dispersion and density imaging (NODDI), which distinguishes between intra-neuronal space, extra-neuronal space, and cerebrospinal fluid and gives information on the coherence of neurite geometries (Zhang et al., 2012). Each of these models yields different measures, named and described in Table 1, that characterize different aspects of water diffusivity and neural microanatomy in the brain.

**TABLE 1:** Diffusion tensor imaging (DTI) and neurite orientation dispersion and density imaging (NODDI) measures, their definitions, and their neurological relevance.

Metric	Definition	Relation to biology
**Diffusion tensor imaging (DTI)**		
Fractional anisotropy (FA)	Measure of the extent to which water diffusion is confined to one direction (Winklewski et al., 2018)	Low values represent water diffusion in many directions (as would occur in cerebrospinal fluid or in dendritic trees); high values represent water diffusion in mostly 1 direction (as would occur in a unidirectional axon bundle) (Wedeen et al., 2005; Morgan et al., 2013)
Mean diffusivity (MD)	Measure of the total diffusion within a voxel (Wen et al., 2019)	High values represent decreased confinement of water molecules (as in regions of low cell or neurite density or regions with tissue damage); low values represent increased confinement of water molecules (as in regions of high cell or neurite density) (Stebbins and Murphy, 2009)
Axial diffusivity (AxD)	Measure of water diffusion along the axis with greatest water diffusion (Winklewski et al., 2018)	Low values represent regions with axonal damage (Winklewski et al., 2018) or areas with few axons or axons that are not coherently organized (Solowij et al., 2017); high values represent regions with dense, coherent fiber tracts
Radial diffusivity (RD)	Measure of the ability of water to diffuse perpendicular to the axis with greatest water diffusion (Winklewski et al., 2018)	High values represent demyelination (Wen et al., 2019) or low axon density or coherence (Solowij et al., 2017); low values represent well-myelinated axons or high axon density or coherence
**Neurite orientation dispersion and density imaging (NODDI)**		
Orientation dispersion (OD)	Measure of relative orientations of neurites in tissue (Zhang et al., 2012)	High values represent neurites that propagate in many different directions relative to one another; low values represent neurites that propagate in the same direction (Zhang et al., 2012)
Isotropic volume fraction (ISOVF)	Measures volume fraction of “free water,” or water with unrestricted diffusivity, in tissue (Zhang et al., 2012)	High values represent low cell density and/or high volumes of cerebrospinal fluid (CSF) (Zhang et al., 2012) and are associated with neurodegeneration (Ogawa et al., 2021); low values represent high cell or neurite density
Intracellular volume fraction (ICVF)	Measures volume of space occupied by neurites (Zhang et al., 2012)	High values represent areas with highly dense neurites; low values represent areas with low neurite density (Zhang et al., 2012)

Previous work has revealed that DTI and NODDI metrics relate to AD/dementia status and cognitive function. Generally, cognitive dysfunction correlates with lower fractional anisotropy (FA) and higher mean diffusivity (MD) (Mayo et al., 2019; Wen et al., 2019; Zavaliangos-Petropulu et al., 2019). Individuals with AD tend to have lower white matter FA and higher MD, axial diffusivity (AxD), and radial diffusivity (RD) than healthy controls (Mayo et al., 2019). Additionally, compared to healthy controls, individuals with AD were found to have lower cortical intracellular volume fraction (ICVF) and orientation dispersion (OD) (Vogt et al., 2020), and individuals with mild cognitive impairment (MCI) were found to have lower white matter ICVF and OD than healthy controls (Fu et al., 2020). Together, these data indicate that AD-related cognitive decline may be mitigated by microanatomical features of the brain.

The relationship between brain microanatomy and cognition may in part explain genetic influences on cognitive outcomes during healthy aging and AD-related cognitive decline. Wang et al. demonstrated that microstructural brain features, as conveyed by DTI, are highly dependent on genetic background. They generated DTI metrics for 322 brain regions in a panel of four genetically diverse mouse strains and found that 298 regions had a significant effect of strain on FA, 128 regions had a significant effect of strain on MD, 117 regions had a significant effect of strain on AxD, and 175 regions had a significant effect of strain on RD. Several regions in this study also had high heritability of FA and MD (Wang et al., 2020). Furthermore, AD-specific studies have reported that mouse models of amyloid pathology, a hallmark of AD (Murphy and LeVine, 2010), exhibited significantly different DTI and NODDI values in memory-relevant regions, including the hippocampus, cingulum, fimbria, and cortical areas (Whittaker et al., 2018; Colon-Perez et al., 2019; Manno et al., 2019; Falangola et al., 2021).

These studies demonstrate that DTI and NODDI measures can represent changes in neural microstructure influenced by amyloid pathology and natural genetic variation. However, knowing how genetic background and amyloid pathology together influence neural microanatomy is necessary to determine if brain microanatomy is a strong, heritable source of brain reserve against AD.

To this end, we have performed DTI and NODDI imaging in genetically diverse, mature adult mice with the amyloid-driving 5XFAD transgene. Specifically, we imaged the F1 progeny of crosses between the 5XFAD transgenic mouse model of AD, maintained on a congenic C57BL/6J background, and five genetically diverse strains (C57BL/6J, DBA/2J, FVB/NJ, CAST/EiJ, and WSB/EiJ). The 5XFAD model was chosen for its early and aggressive amyloid pathology, which can begin at 1.5 months of age, and cognitive impairment, which begins at 6–8 months of age (Forner et al., 2021; Oblak et al., 2021). To capture neurological features that may contribute to brain reserve, we performed imaging at 3 months of age, at which mice are expected to be mature young adults (Flurkey et al., 2007), but prior to expected onset of cognitive senescence. At 5 months of age, during which non-transgenic (Ntg) mice are still considered to be in early adulthood and are not expected to exhibit differences in health and microstructure from 3 months of age (Flurkey et al., 2007; Hammelrath et al., 2016; Kerkenberg et al., 2021), mice underwent contextual fear conditioning (CFC) to assess the impacts and interactions of strain, sex, and the 5XFAD transgene on cognitive health and to investigate relationships between brain microstructure and memory.

Our findings revealed that strain, more than sex and the 5XFAD transgene, influences microstructural brain features in 3-month-old mice and baseline freezing and contextual fear memory (CFM) performance in 5-month-old mice. Our results recapitulated findings demonstrating the brain-wide impacts of strain on neural microanatomy as conveyed by dMRI and demonstrated that the 5XFAD transgene does not strongly alter early brain microstructure. Additionally, our work revealed that strain, sex, and the 5XFAD transgene interact to influence memory in genetically diverse mice, and we suggest relationships between memory health and microstructure of several brain regions in Ntg mice. Our results demonstrated the value of multimodal dMRI imaging in inferring brain microstructure and in uncovering the relationship between genetics, brain microstructure, and cognitive health.

## 2. Materials and methods

### 2.1. Experimental subjects

All experiments described herein comply with the NIH Office of Laboratory Animal Welfare D16-00170, AUS #16040, and JAX AAALAC accreditation #000023. The welfare of all experimental subjects was assessed routinely.

**FIGURE 1:**
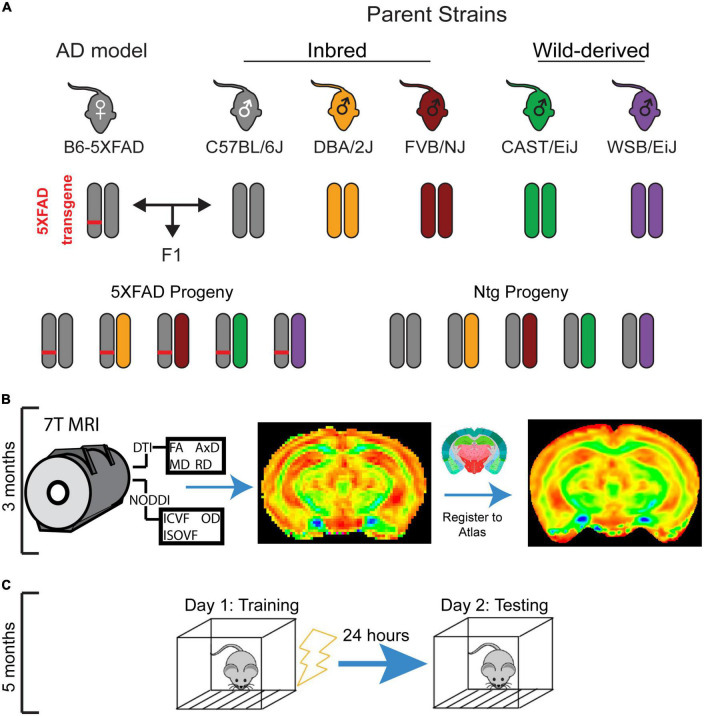
Experimental mouse panel and timeline. **(A)** Breeding scheme for mouse panel. The 5XFAD mouse model of Alzheimer’s disease (AD), which has five causal human AD mutations, maintained on a congenic C57BL/6J background was crossed to DBA/2J, FVB/NJ, WSB/EiJ, CAST/EiJ, and C57BL/6J mice. Transgenic and non-transgenic F1 progeny of both sexes were used in this study. **(B)** Mice underwent diffusion tensor imaging (DTI) and neurite orientation dispersion and density imaging (NODDI) imaging at 3 months of age. **(C)** At 5 months of age, mice underwent contextual fear conditioning, which involves a training session in which mice receive four footshocks in a novel environment, followed by a testing session 24 h later to assess fear memory.

This experiment utilized the F1 progeny of males from five genetically diverse mouse strains (C57BL/6J, DBA/2J, FVB/NJ, CAST/EiJ, and WSB/EiJ) and C57BL/6J female mice hemizygous for the 5XFAD AD transgene (Figure 1A). The 5XFAD transgene drives expression of two human proteins with AD-related mutations: the amyloid precursor protein (APP) with the Florida (I716V), London (V717I), and Swedish (K670N/M671L) mutations and the presenilin 1 protein (PSEN1) harboring the M146L and L286V mutations (Oakley et al., 2006).

Strains crossed to the C57BL/6J-5XFAD mouse to generate this mouse panel are described below:

1. C57BL/6J (B6) (JAX stock #000664) is the strain of the maternal parent that gave rise to the BXD mouse panel, a genetically diverse panel used to enhance mouse genetic mapping studies (Peirce et al., 2004).
2. DBA/2J (D2) (JAX stock #000671) is the paternal parent that gave rise to the BXD mouse panel (Peirce et al., 2004).
3. FVB/NJ (JAX stock #001800) is an albino inbred mouse strain which is well-known for its vulnerability to the Friend leukemia virus B and histamine disphosphate (Wong et al., 2012).
4. CAST/EiJ (JAX stock #000928) is a wild-derived inbred mouse strain originating from the *Mus musculus castaneus* subspecies located in Southeast Asia (Timmermans et al., 2017). These mice tend to have smaller bodies than other common mouse strains (Wang et al., 2020).
5. WSB/EiJ (JAX stock #001145) is a wild-derived inbred strain whose founders were obtained in Eastern Shore, Maryland.

These strains were selected because of their genetic diversity (Keane et al., 2011; Onos et al., 2019; Ayme-Dietrich et al., 2021) and because, when these strains harbor transgenes to induce Alzheimer’s-related pathology, they differ in AD-related phenotypes, including pathology, longevity, and susceptibility to neuron loss (Neuner et al., 2019; Onos et al., 2019; Swarup et al., 2019).

From these crosses, 96 F1 transgenic and Ntg progeny of both sexes were generated and used in this study. To determine the impact of mouse strain and the 5XFAD transgene on brain microstructure, all mice underwent MRI imaging at 3 months of age (Figure 1B). 2 months later, after it was evident that strain influenced brain microstructure, animals underwent CFC at 5 months of age (Figure 1C) to investigate the relationships between strain, sex, 5XFAD genotype, and learning and memory. Between 3 and 6 months of age, mice are considered mature adults, comparable to humans at 20–30 years of age. During this age range, CFC performance (data unpublished), DTI values, volumes, and myelination in regions throughout the mouse brain, as well as general health and mouse survivability, have been demonstrated not to vary notably in Ntg mice (Flurkey et al., 2007; Hammelrath et al., 2016; Kerkenberg et al., 2021). Therefore, our study design enabled us to capture cognition and microstructure in mature adult mice and had the added benefit of allowing mice to recover from anesthesia during imaging, which may affect cognitive performance (Liu et al., 2014). Group numbers for strain, sex, and 5XFAD genotype are outlined in Table 2. All animals were maintained under a 12-h light/dark cycle, housed in pens of two-five mice, and given *ad libitum* access to the LabDiet 5K0G food pellet and water.

**TABLE 2:** Strain/sex/5XFAD genotype groups were well balanced in terms of size.

Strain	Sex	5XFAD Genotype	Sample size *n*
B6	F	Ntg	5
B6xD2	F	Ntg	5
B6xCAST	F	Ntg	5
B6xFVB	F	Ntg	5
B6xWSB	F	Ntg	5
B6	M	Ntg	5
B6xD2	M	Ntg	5
B6xCAST	M	Ntg	3
B6xFVB	M	Ntg	5
B6xWSB	M	Ntg	5
B6	F	5XFAD	5
B6xD2	F	5XFAD	5
B6xCAST	F	5XFAD	5
B6xFVB	F	5XFAD	5
B6xWSB	F	5XFAD	5
B6	M	5XFAD	5
B6xD2	M	5XFAD	5
B6xCAST	M	5XFAD	3
B6xFVB	M	5XFAD	5
B6xWSB	M	5XFAD	5

Group sizes for all strain/sex/5XFAD genotype groups utilized in this study are detailed above. F, female, M, male, Ntg, non-transgenic, 5XFAD, 5XFAD transgene carrier.

### 2.2. Image acquisition

Our image acquisition protocol was adapted from a previous mouse NODDI study (Colon-Perez et al., 2019). All images were acquired using a 7.0T MRI (BioSpec 70/20 USR MRI scanner, Bruker BioSpin Corporation, Billerica, MA). An 86 mm Bruker volume coil was used as the excitation radiofrequency (RF) coil, while a Bruker 2 × 2 20 mm Phase Array coil was used as the receiving coil. For both DTI and NODDI imaging, a 4-shot spin-echo echo planar imaging (SE-EPI) sequence built into the Bruker BioSpin scanner was used (TR = 4000 ms, TE = 19.54 ms, repetition number = 1; number of averages = 4; gradient duration = 4 ms; diffusion time spacing = 10 ms; bandwidth = 300 KHz; FOV = 13.12 × 15.75 mm^2^; MS = 80 × 96; in-plane resolution: 164 × 164 μm; slice thickness = 750 μm; slice number/mouse: 17, without gaps between slices; phase encoding direction: ventral to dorsal). Fat suppression and 3-band saturations were used to reduce signals from non-brain tissue. Signal-to-noise ratios (SNR) were calculated separately for dorsal and ventral segments of our brain images because MRI signals tend to be stronger closer to the phase array coil, which was situated more closely to the dorsal aspect of mouse brains during imaging. Two regions were drawn in the dorsal and ventral portions of mouse brain images (Supplementary Figure 1A), and SNR for these regions were calculated. The SNR values for the dorsal/ventral regions are as follows: 14.7/5.2 for anatomical images; 56.0/22.4 for *b* = 0 sec/mm^2^ diffusion images; and 26.4/9.4 for *b* = 1200 sec/mm^2^ diffusion images. The following parameters were used for T^2^-weighted imaging: TR = 4000 ms; TE = 45 ms; number of averages = 4; bandwidth = 300 KHz; FOV = 13.12 × 15.75 mm^2^; MS = 80 × 96; in-plane resolution: 82 × 82 μm; slice thickness = 750 μm, slice number/mouse: 17, without gaps between slices; phase encoding direction: ventral to dorsal.

For DTI imaging, we obtained one shell consisting of one baseline B_0_ image (*b* value = 0 sec/mm^2^) and 46 gradient directions (*b* value = 1200 sec/mm^2^), resulting in 47 total images/brain. For NODDI imaging, we acquired another shell corresponding to a b value of 600 sec/mm^2^, with six gradient directions and a baseline B_0_ image. Thus, an additional seven images were obtained for each brain following our NODDI protocol, resulting in 54 NODDI images/brain. Lastly, echo-planar imaging (EPI) blip-up and blip-down scans were performed to enable FSL TOPUP calibration using a reversible pulse sequence created by Dr. Matthew Budde, University of Wisconsin Medical College.^1^

Prior to being situated in the Bruker platform, each mouse was weighed, and anesthesia was induced using 2% isoflurane. Then, the mouse was placed in the Bruker animal platform, and the mouse’s head was held still by a teeth bar, two ear pins, and a nosecone to minimize motion artifacts. During image acquisition, the mouse received air at a flow rate of 130 ml/min with 0.9–1.2% isoflurane through the nosecone to maintain anesthesia, and mouse respiratory rate was maintained at 50–100 breaths per minute. Body heat was maintained at 95.5 ± 0.1°F using a water-heated mouse bed designed and 3D-printed in-house, and mouse vitals were monitored *via* pulse oximetry (SA Instruments, Inc., Stony Brook, NY, USA). After mouse positioning, shimming was performed, and slices were aligned to the coronal mouse brain plane. Image acquisition began with a 10-min resting-state functional MRI protocol (results not presented herein), followed by the multi-shell dMRI protocol (∼57.5 min). The imaging protocol finished with T_2_-weighted anatomical imaging, and each mouse was scanned and maintained under anesthesia for ∼120 min.

### 2.3. Image pre-processing

Bruker RAW MRI datasets were converted into NIFTI file format using a free software package, namely, bruker2nifti.^2^ Raw DTI and NODDI images were manually segmented using ImageJ (Schindelin et al., 2012) and automatically corrected for eddy currents using the *eddy* tool in FSL. Then, DTI and NODDI images were visually inspected in ImageJ. Any frames with severe motion artifacts and their corresponding b values and b vectors were manually removed from the dataset using DSI-Studio.^3^

For DTI, 47 raw image volumes (for 46 gradient directions for a b value of 1200 sec/mm^2^ and one B_0_ image) were obtained. After eddy current and manual motion correction, these images were processed using the FDT Diffusion tool in FSL^4^ to generate maps for FA, MD, RD, and AxD (with MD, RD, and AxD having units of mm^2^/sec). Afterward, all signals not from the brain were removed using the Brain Extraction Tool in FSL and a hand-drawn mask created in ImageJ.

For NODDI, we adapted the protocol utilized in Colon-Perez et al. (2019) and thus obtained 54 raw image volumes (one B_0_ image and six gradient directions for a b value of 600 sec/mm^2^, and one B_0_ image and 46 gradient directions for a b value of 1200 sec/mm^2^). OD, ICVF, and ISOVF maps were generated from eddy current- and manually motion-corrected raw images using the NODDI AMICO python software package, which can be found on GitHub.^5^

### 2.4. Label registration

Feature-based registration was used to fit anatomical, DTI, and NODDI images to our reference atlas, a twice-downsampled version of the P56 Allen Brain Atlas (Allen Institute for Brain Science, 2011). T2-weighted anatomical images underwent linear registration to the reference atlas. This was done to assist in registration of DTI and NODDI images: The automated registration steps used for this study (see below) align brain features, the visual boundaries between brain regions, from raw diffusion images to the same brain features within the reference atlas. Because of differences in feature resolution between FA images and the reference atlas, FA images underwent linear registration to registered T2-weighted anatomical images for corresponding mice prior to feature-dependent non-linear registration to the reference atlas. MD, AxD, RD, ICVF, ISOVF, and OD images had poorer feature clarity than FA images. Therefore, the linear transformations used for FA images were applied to the other raw DTI and NODDI images during linear registration to the registered anatomical image for corresponding mice. These transformed images were then non-linearly registered to the transformed anatomical image. Once transformed DTI and NODDI images were obtained, a common mask was applied to all images to ensure that these images only contained data confined to the same reference space. For each dMRI image registered thus, one dMRI value was generated per region of interest (ROI) *via* the FSL *fslstats* function, which applies labels from a reference atlas annotation file to our dMRI images and then performs statistics on voxels within each labeled ROI. Using *fslstats*, we calculated the mean voxel intensity for all voxels within an ROI from each hemisphere. In this work, we refer to these mean ROI voxel intensities as ROI values, on which all statistics presented herein are performed. All linear registration steps involved the trilinear interpolation method and were completed using the FSL *flirt* function. Non-linear feature registration was implemented using ANTs (*antsRegistrationSyNQuick.sh* function).

### 2.5. Contextual fear acquisition and memory paradigms

Mice were given 2 months to recover from stress and anesthesia, which may influence cognitive performance (Liu et al., 2014), involved in image acquisition prior to assessment of cognitive health. Mice then underwent a contextual fear acquisition (CFA) and CFM paradigm. In the CFA paradigm, one mouse was inserted into a novel environment, a fear conditioning box, for 10 min. In the first 2 min of this session, the mouse was not shocked, so any freezing occurring in this session was not due to foot shocks administered during the session. The percentage of time spent freezing during this initial 2-min period was referred to as the mouse’s baseline freezing. Each mouse received a foot shock 2, 4, 6, and 8 min into the session. Each foot shock marked the beginning of a new 2-min bin during the session; the final four bins were referred to as CFA Post-shock interval 1 (PS1), PS2, PS3, and PS4, numbered in chronological order.

The CFM paradigm occurred 24 h after the CFA paradigm. During this session, the mouse was inserted for 10 min into the same box in which CFA occurred, but no foot shocks were delivered. Percentage of time freezing was measured across this 10-min session and referred to as CFM percent freezing.

For both CFA and CFM, freezing was measured using a video analysis software called FreezeFrame 5 (©2022 Actimetrics). Briefly, this software tracks motion by analyzing the number of pixels that change from frame to frame. The software user manually adjusted a freezing “threshold,” the number of pixels required to change between frames for the software to classify motion. Then, the software yielded percent of time freezing for all CFA bins and for the total CFM session.

### 2.6. Statistics

For all DTI and NODDI measures, brain volumes were segmented into 83 ROIs as described above. The values of all voxels in each ROI from both hemispheres were averaged to give one value per ROI.

Type III, 3-way ANOVAs testing for effects of strain, sex, and 5XFAD genotype on each dMRI measure in each ROI were performed. All *p*-values obtained thus were false discovery rate (FDR)-adjusted using the Benjamini-Hochberg correction. ROIs with five or more dMRI readouts with significant effects of strain were previously implicated in AD or healthy aging and were therefore selected for *post hoc* analyses. Specifically, Tukey’s honest significance test was performed on a Type I ANOVA model testing for an effect of strain on each dMRI measure shown to have a significant strain effect with the Type III ANOVAs described above. All *p*-values generated from Tukey tests were FDR-adjusted using the Benjamini-Hochberg correction. With the exception of the lateral septal complex, dorsal hippocampal commissure, and behavioral-state related pons, RD and AxD values were omitted from these analyses because their strain differences were very similar to MD. This is expected, as MD is the average of RD and AxD. For the lateral septal complex, dorsal hippocampal commissure, and behavioral state-related pons, AxD and RD strain differences did not strongly parallel those observed for MD. Therefore, *post hoc* analyses were performed for AxD and RD readouts in these three ROIs.

Heritability of strain means (*h*^2^_*rix*_) for all mice examined herein were calculated with the following formula:


h2rix=1N∑i=1NVg,i(Vg,i+(Ve,i/ni¯))

where *i* refers to a sex/5XFAD genotype group, *N* is the number of sex/5XFAD genotype groups (4 for our study), *V*_*g*_ is the variance explained by genetics (the variance across strain averages for a particular sex/5XFAD genotype group), *V*_*e*_ is the variance explained by environmental factors (the mean within-strain variance for a particular sex/5XFAD genotype group), and n̄ is the average number of mice/strain for a particular sex/5XFAD genotype group. The *h*^2^_*rix*_ enabled us to examine the variation in dMRI measures explained by the differential genomes of the strains under investigation. However, it should be noted that *h*^2^_*rix*_ will increase with increasing values of n̄. The mean *n* value for each strain/sex/5XFAD genotype group in this study was 4.28, and individual *n* values for each strain/sex/5XFAD genotype group is presented in Table 2.

Independent 3-way Type III ANOVAs testing for effects of strain, sex, and 5XFAD genotype were applied to baseline freezing, CFA PS4 percent freezing, and CFM percent freezing. One Benjamini-Hochberg correction was applied to the *p*-values generated from both the baseline freezing and CFM percent freezing ANOVAs because only baseline freezing and CFM exhibited significant effects of strain, sex, 5XFAD genotype, and/or interactions thereof (see Table 3). Then, one-way ANOVAs testing for effects of strain on baseline freezing and CFM percent freezing were performed, followed by Tukey’s honest significance tests to evaluate strain differences in baseline freezing and CFM. All *p*-values from baseline freezing and CFM Tukey’s honest significance tests were corrected for multiple comparisons using the Benjamini-Hochberg method.

**TABLE 3:** Strain, sex, and 5XFAD genotype exhibited significant effects on baseline freezing and CFM.

	Sum of squares	*F* value	Nominal *P*-value	FDR-adjusted *P*-value
**Effects of strain, sex, and 5XFAD genotype on baseline freezing**				
Strain	**188**.**0**	**9.18**	**0.00**	**0.00**
Sex	**37**.**1**	**7.24**	**0.01**	**0.03**
Genotype	2.2	0.43	0.51	0.65
Strain*Sex	**105**.**3**	**5.14**	**0.00**	**0.00**
Strain*Genotype	29.4	1.44	0.23	0.43
Sex*Genotype	1.5	0.30	0.59	0.70
Strain*Sex*Genotype	4.7	0.23	0.92	0.92
Residuals	332.9			
**Effects of strain, sex, and 5XFAD genotype on CFA PS4 percent freezing**				
Strain	1288.9	0.55	0.70	0.80
Sex	348.1	0.59	0.45	0.63
Genotype	2345.2	3.97	0.05	0.12
Strain*Sex	966.3	0.41	0.80	0.84
Strain*Genotype	1080.7	0.46	0.77	0.84
Sex*Genotype	593.8	1.01	0.32	0.51
Strain*Sex*Genotype	2049.1	0.87	0.49	0.65
Residuals	38354.3			
**Effects of strain, sex, and 5XFAD genotype on CFM percent freezing**				
Strain	**11539**.**9**	**9.16**	**0.00**	**0.00**
Sex	275.6	0.87	0.35	0.53
Genotype	1070.0	3.40	0.07	0.15
Strain*Sex	**4895**.**7**	**3.89**	**0.01**	**0.02**
Strain*Genotype	2617.6	2.08	0.09	0.19
Sex*Genotype	**1900**.**2**	**6.03**	**0.02**	**0.04**
Strain*Sex*Genotype	1538.9	1.22	0.31	0.51
Residuals	20473.6			

No significant effects of strain, sex, or 5XFAD genotype on contextual fear acquisition (CFA) PS4 were observed. Values were derived from Type III, 3-way ANOVAs testing for effects of sex, strain, and 5XFAD genotype on baseline freezing, CFA PS4 percent freezing, and contextual fear memory (CFM) percent freezing. FDR-correction only included correction for *P*-values derived from ANOVAs on baseline freezing, CFA PS4 percent freezing, and CFM percent freezing. Bold values represent significant effects after FDR adjustment.

## 3. Results

### 3.1. Strain exhibited a significant effect on dMRI measures in the most ROIs and was especially influential in AD- and memory-relevant ROIs regardless of 5XFAD genotype

**FIGURE 2:**
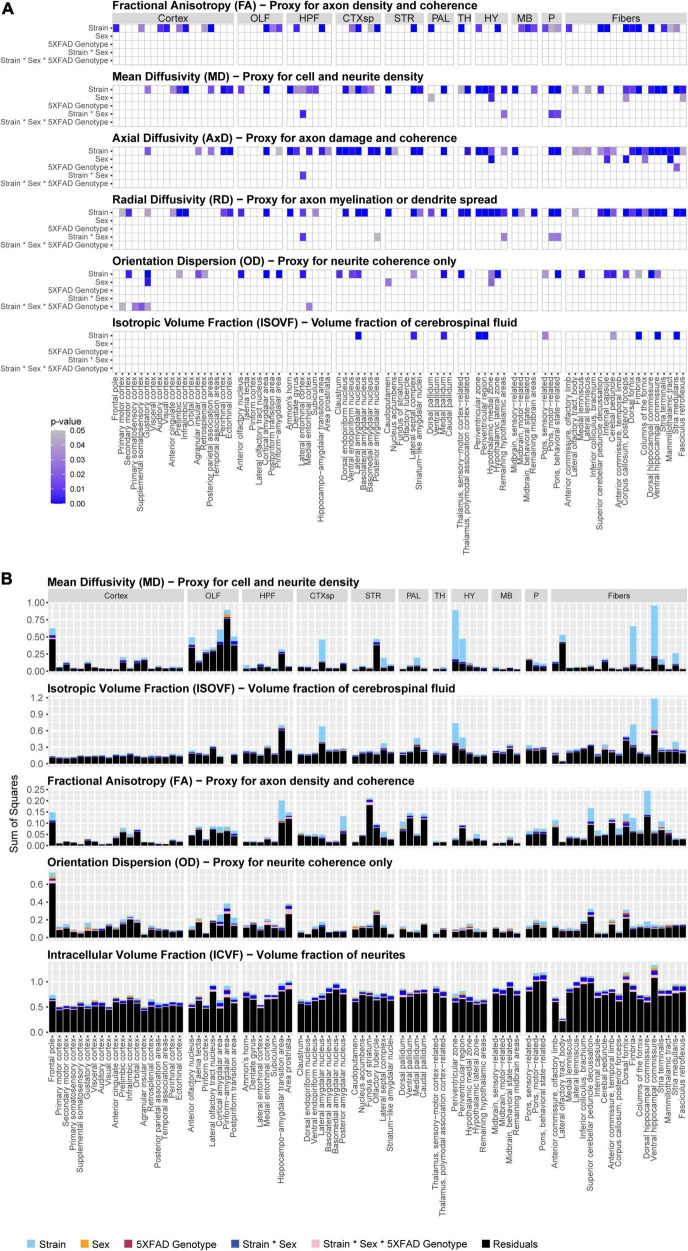
Significance and effect sizes of strain, sex, and 5XFAD genotype main effects and interactions on brain microanatomy related diffusion magnetic resonance imaging (dMRI) measures. **(A)** Summary of main effects and interactions of strain, sex, and 5XFAD genotype on brain microanatomy-related dMRI measures. All *p*-values are corrected for multiple comparisons (Type III ANOVA, Benjamini-Hochberg-corrected). White spaces represent non-significant (*p*_corrected_ > 0.05) effects. Intracellular volume fraction (ICVF) effects were omitted from the figure because none were significant. The following abbreviations of large region names were used as facet labels in all faceted figures: “Cortex,” isocortex; “OLF,” olfactory areas; “HPF,” hippocampal formation; “CTXsp,” cortical subplate; “STR,” striatum; “PAL,” pallidum; “TH,” thalamus; “HY,” hypothalamus; “MB,” midbrain; “P,” pons; “Fibers,” fiber tracts. **(B)** Strain strongly influences microstructure-related dMRI features in 3-month-old mice. Presented are sums of squares as calculated in a three-way Type III ANOVA testing for effects of strain, sex, and 5XFAD genotype. All dMRI values were scaled for this figure such that the median value for each dMRI in each region ranged between 0.1 and 1. Alzheimer’s disease (AD)- and memory-relevant regions of interest (ROIs) showed high effect sizes (sums of squares) of strain. Strain explains more variance than sex, 5XFAD genotype, or interaction terms on neural dMRI measures, *p*-values and sums of squares of sex*5XFAD genotype and strain*5XFAD genotype interactions were omitted from the figure because none were significant. Additionally, axial diffusivity (AxD) and radial diffusivity (RD) sums of squares closely paralleled those observed for mean diffusivity (MD) values, so they were omitted from this figure to enhance clarity.

In this study, we sought to determine if brain microanatomy could be a heritable source of brain reserve against AD. To answer this question, we first determined how brain microanatomy-related dMRI measures varied across strain, sex, and 5XFAD genotype. To do so, five genetically diverse mouse strains were crossed to the 5XFAD mouse model of AD maintained on a congenic C57BL/6J background. 96 mature, young adult F1 progeny from these crosses, including male and female carriers and non-carriers of the 5XFAD transgene, were used in this experiment. At 3 months of age, these mice underwent DTI and NODDI imaging. Main effects of strain (here, strain refers to mice produced by specific crosses performed for this study, i.e., B6, B6xD2, B6xFVB, B6xCAST, or B6xWSB), sex, 5XFAD genotype, and interactions thereof, on four DTI measures and three NODDI measures (see Table 1 for more information) in each of 83 ROIs were analyzed. Significant main effects and interactions for each of the dMRI measures in each ROI are summarized in Figure 2A. No significant effects were observed for ICVF values obtained in any ROI, and no significant sex*5XFAD genotype or strain*5XFAD genotype interactions were observed.

Of effects of strain, sex, and 5XFAD genotype, effects of strain were by far the most frequent. dMRI measures with significant effects of strain were observed in 70 ROIs. On the other hand, dMRI measures with significant effects of sex, 5XFAD genotype, sex*strain interactions, and strain*sex*5XFAD genotype interactions were observed in only nine, one, five, and five ROIs, respectively (Figure 2A).

Importantly, strain explained a large amount of variance in ROIs in the cortical subplate, hypothalamus, hippocampal formation, and fiber tracts (Figure 2B), four brain areas highly relevant to memory and AD (Hyman et al., 1984; Simić et al., 1997; Amorapanth et al., 2000; Giannakopoulos et al., 2003; Maren and Quirk, 2004; Bird and Burgess, 2008; Castaño et al., 2013; Butler et al., 2018; Colon-Perez et al., 2019; Liao et al., 2021; Jordan et al., 2022; Tsui et al., 2022), regardless of 5XFAD transgenic status.

**FIGURE 3:**
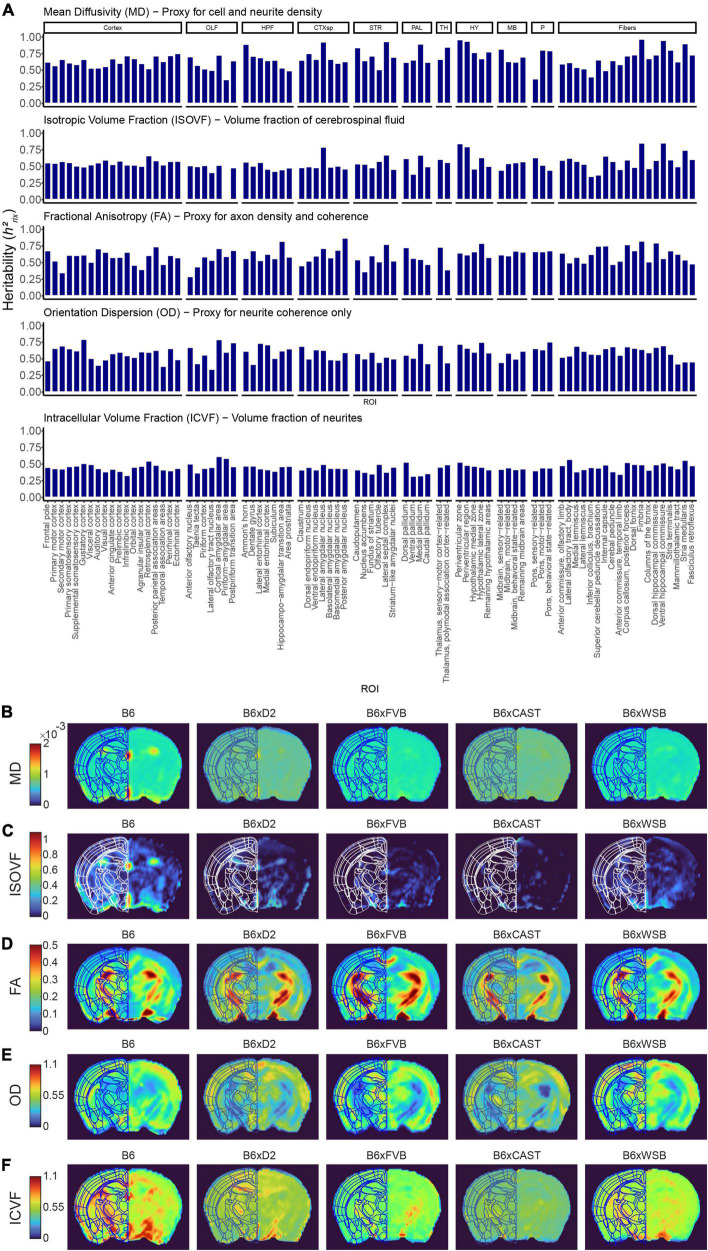
Strain strongly influences microstructure-related dMRI features in 3-month-old mice. **(A)** dMRI measures in Alzheimer’s disease (AD)- and memory-relevant ROIs showed high heritability, indicating the effect of natural genetic variation (strain) on brain microstructure. Presented are *h*^2^_*rix*_ values, which represent the heritability of strain means. AxD and RD values paralleled MD values, so AxD and RD *h*^2^_*rix*_ values were omitted from this figure to enhance clarity. **(B–F)** Representative images of MD **(B)**, isotropic volume fraction (ISOVF) **(C)**, fractional anisotropy (FA) **(D)**, orientation dispersion (OD) **(E)**, and intracellular volume fraction (ICVF) **(F)**, one from each strain examined, shown to demonstrate robust strain differences existing among DTI and NODDI measures.

To more directly examine the influence of natural genetic variation on brain microstructure, we calculated the heritability (*h*^2^_*rix*_) of dMRI measure strain means (Figure 3A and Supplementary Figure 2D). Our results indicated heritabilities comparable to those exhibited in higher-resolution DTI studies, which again suggests that effects of natural genetic variation on brain microstructure are robust (for representative images of dMRI strain differences, see Figures 3B–F and Supplementary Figures 2E, F).

Some ROIs were observed to have many dMRI measures with significant effects of strain, suggesting the unique sensitivity of their microanatomy to genetic variation. Of note, all ROIs with five or more dMRI measures with significant strain effects have been previously implicated in AD or age-related cognitive changes. These included white matter and gray matter ROIs distributed throughout the forebrain and hindbrain, suggesting widespread effects of natural genetic variation on neural microstructure. ROIs with five or more strain-influenced dMRI measures included the dorsal hippocampal commissure, ventral hippocampal commissure, fimbria, and stria medullaris in brain white matter; the cortical amygdalar area, lateral amygdalar nucleus, lateral septal complex, and medial pallidum in the forebrain gray matter; and periventricular zone of the hypothalamus (periventricular zone) and behavioral state-related pons in the hindbrain gray matter.

To probe strain differences in microanatomy in these ROIs, *post hoc* analyses were performed. For each dMRI measure besides RD and AxD, in each of the above 10 ROIs that had a significant effect of strain, a one-way, Type I ANOVA was conducted that tested for effects of strain on that measure in each of these 10 ROIs. The lateral septal complex, dorsal hippocampal commissure, and behavioral state-related pons were the only ROIs with RD and AxD values on which *post hoc* analyses were performed (Supplementary Figures 2A–C). For all other ROIs, MD, AxD, and RD measurements showed highly similar strain differences, so AxD and RD values were excluded from the analysis (see Section “2 Materials and methods”). These strain differences are explained below.

### 3.2. Multiple dMRI metrics suggested strain-driven differences in axon density and organization in four white matter tracts

#### 3.2.1. Stria medullaris

**FIGURE 4:**
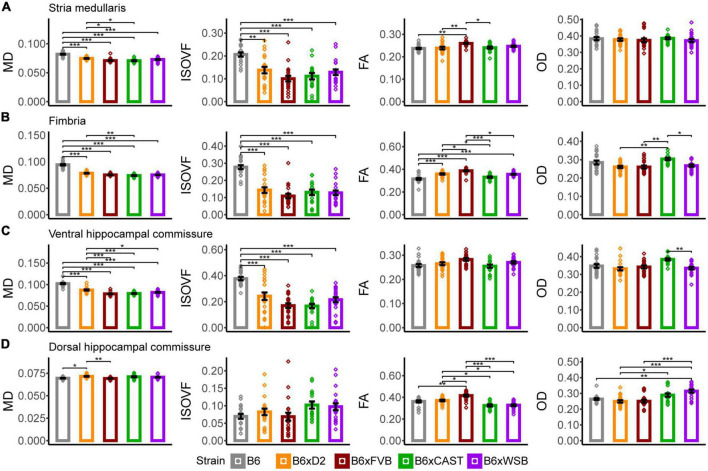
Stria medullaris, fimbria, ventral hippocampal commissure, and dorsal hippocampal commissure white matter organization, as conveyed by dMRI readouts, was especially sensitive to strain. MD, ISOVF, FA, and OD strain differences in the stria medullaris **(A)**, fimbria **(B)**, ventral hippocampal commissure **(C)**, and dorsal hippocampal commissure **(D)**. Bars represent data from 5XFAD and (Ntg) animals collapsed together and grouped by strain because no 5XFAD genotype effects were observed, with the exception of the stria medullaris. Bar color corresponds to strain. Bar heights represent strain means. Error bars represent standard error on the mean. Significance was determined by Tukey’s honest significance test following one-way ANOVAs testing for effects of strain on dMRI metrics (see Section “2 Materials and methods”), with resulting *p*-values false discovery rate (FDR)-adjusted (*p_*corrected*_ < 0.05; **p_*corrected*_ < 0.01; ***p_*corrected*_ < 0.001).

B6 mice had significantly higher MD and ISOVF values in the stria medullaris than the progeny of all other crosses (Figure 4A). Additionally, B6xD2 mice had significantly greater MD values than B6xCAST and B6xFVB mice, and B6xFVB FA values were significantly higher than those of B6, B6xD2, and B6xCAST mice. Given that the stria medullaris is a white matter tract and that no strain differences in OD were observed, these differences were likely due to differences in myelination and axon density.

#### 3.2.2. Fimbria

B6 mice had significantly higher MD than the progeny of all other crosses, and B6xD2 mice had significantly higher MD than B6xCAST mice (Figure 4B), suggesting strain differences in axon volume, density, or coherence. Additionally, B6 mice had significantly higher ISOVF than the progeny of all other crosses in this study, suggesting reduced volume fraction of axons and increased volume fraction of CSF in B6 mice in this white matter ROI. A higher volume of CSF in this white matter tract may also explain why B6 mice were observed to have significantly lower FA values than B6xD2, B6xFVB, and B6xWSB mice. B6xFVB mice had significantly higher FA than the progeny of all other crosses, suggesting that the fimbria in B6xFVB mice is uniquely coherent.

B6xCAST mice had significantly higher OD than B6xD2, B6xFVB, and B6xWSB mice, suggesting that axons are less coherently organized in this fiber bundle in B6xCAST mice than B6xD2, B6xFVB, and B6xWSB mice.

#### 3.2.3. Ventral hippocampal commissure

In the ventral hippocampal commissure, B6 mice had significantly higher MD and ISOVF values than the progeny of all other crosses (Figure 4C), again suggesting high CSF content and low axon coherence or density in B6 mice. Additionally, B6xD2 mice had significantly higher MD values than B6xFVB, B6xCAST, and B6xWSB mice, but B6xD2 ISOVF values were only significantly different from B6 mice. Therefore, differences in MD between B6xD2 mice and B6xCAST, B6xFVB, and B6xWSB mice are likely due to differences in axon coherence or myelination.

B6xFVB had higher FA than the progeny of all other crosses, although these differences did not reach significance. In addition, B6xCAST mice had significantly higher OD values than B6xWSB mice, suggesting greater axon coherence among B6xWSB mice than B6xCAST mice.

#### 3.2.4. Dorsal hippocampal commissure

In the dorsal hippocampal commissure, B6xD2 mice had significantly higher MD values than B6 and B6xFVB mice (Figure 4D); significantly higher AxD than B6xCAST and B6xWSB mice; significantly higher RD than B6xFVB mice; and significantly lower RD than B6xCAST and B6xWSB mice (Supplementary Figure 2A). Taken together, MD and RD differences between B6xD2 and B6xFVB imply lower myelination in B6xD2 mice than B6xFVB mice, and AxD and RD differences between B6xD2, B6xCAST, and B6xWSB mice reflect greater myelination and axon coherence in B6xD2 mice than B6xCAST and B6xWSB mice. No significant ISOVF differences were observed among mice in this ROI.

B6xFVB mice had significantly higher FA values than the progeny from all other crosses, and B6xD2 mice had significantly higher FA than B6xCAST and B6xWSB mice. B6xD2 mice also had significantly lower OD than B6xCAST mice, and B6xWSB mice had significantly higher OD than B6xD2, B6, and B6xFVB mice. These results suggested uniquely high dorsal hippocampal commissure axon coherence in B6xFVB mice, with B6xD2 mice also having relatively high axon coherence in the dorsal hippocampal commissure. On the other hand, B6xWSB mice, as indicated by their high OD and low FA values, again seem to have less coherent axon organization in the dorsal hippocampal commissure than the progeny of other crosses in this cohort.

### 3.3. Multiple dMRI metrics suggested strain-driven differences in CSF content, cellular density, and neurite organization in four forebrain gray matter ROIs

#### 3.3.1. Cortical amygdalar area

**FIGURE 5:**
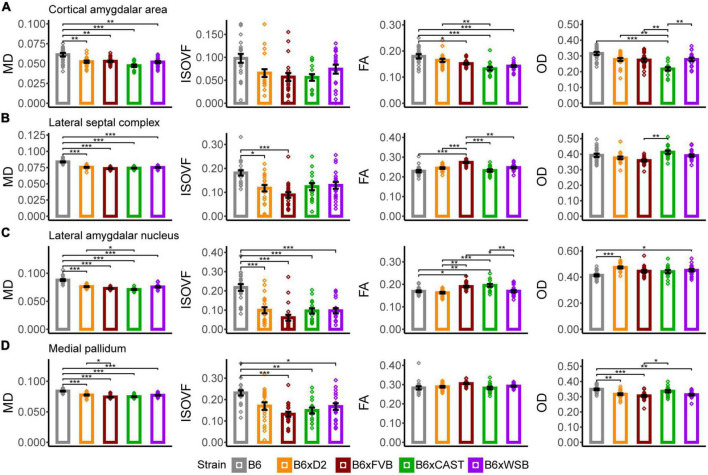
The cortical amygdalar area, lateral septal complex, lateral amygdalar nucleus, and medial pallidum were the forebrain gray matter ROIs whose microstructure, as conveyed by dMRI readouts, was most sensitive to strain. MD, ISOVF, FA, and OD strain differences in the cortical amygdalar area **(A)**, lateral septal complex **(B)**, lateral amygdalar nucleus **(C)**, and medial pallidum **(D)** suggested strain-dependent differences in forebrain gray matter neurite organization, cell density, and cerebrospinal fluid (CSF) content in 3-month-old mouse brains. Bar color corresponds to strain. Bar heights represent strain means. Bars represent data from 5XFAD and Ntg animals collapsed together and grouped by strain because no 5XFAD genotype effects were observed. Error bars represent standard error on the mean. Significance was determined by Tukey’s honest significance test following one-way ANOVAs testing for effects of strain on dMRI metrics (see Section “2 Materials and methods”), with resulting *p*-values FDR-adjusted (*p_*corrected*_ < 0.05; **p_*corrected*_ < 0.01; ***p_*corrected*_ < 0.001).

In the cortical amygdalar area, B6 mice had significantly higher MD values than the progeny of all other crosses in our mouse cohort (Figure 5A), indicating that B6 mice have lower cell or axonal density in this region. B6 FA was significantly higher than B6xFVB, B6xCAST, and B6xWSB mice, while FA in B6xD2 mice was significantly higher compared to B6xCAST mice. These FA values suggested relatively high neurite coherence in the cortical amygdalar area of B6 and B6xD2 mice relative to the progeny of other crosses studied herein. B6xCAST mice exhibited significantly lower OD values than the progeny of other crosses. It is notable that B6 mice had higher FA and OD values than B6xCAST, as high FA suggests high neurite coherence, while high OD suggests low neurite coherence. We may speculate that in the cortical amygdalar area, the relatively high MD, FA, and OD in B6 could be explained by a lower cell density in this ROI, along with a compensatory increase in olfactory bulb afferent axon density (Root et al., 2014) and cortical amygdalar area dendritic arborization. However, follow-up immunohistochemical studies are necessary to confirm this notion.

#### 3.3.2. Lateral septal complex

B6 mice had significantly higher MD (Figure 5B), AxD, and RD (Supplementary Figure 2B) values than the progeny of all other crosses in this study. Notably, the mean MD of B6 was especially high, even for other areas of the brain (Supplementary Figure 3) and seemed to be driven by high AxD values. Therefore, MD values may represent higher density and coherence of axons projecting from the lateral septal complex (Deng et al., 2019) in B6 mice than the progeny of all other crosses studied herein. However, ISOVF values were significantly higher in B6 mice than in B6xD2 and B6xFVB mice, suggesting that, amidst higher axon density, B6 mice have fewer cells or neurites than B6xD2 and B6xFVB mice. Together, MD and ISOVF strain differences suggested higher CSF content, lower cell density, and/or higher axon coherence in the lateral septal complex of B6 mice than in the progeny of other crosses.

In addition, B6xFVB mice had significantly lower RD and significantly higher FA in the lateral septal complex than the progeny of all other crosses, suggesting high axon coherence and/or myelination in B6xFVB mice. Importantly, only B6xCAST mice had significantly higher OD values than B6xFVB mice. This suggests that strain differences in lateral septal complex FA and RD signals can be explained by increased axon myelination, which tends to reduce RD values (Wen et al., 2019), and not neurite coherence.

#### 3.3.3. Lateral amygdalar nucleus

B6xD2 mice had significantly higher MD but significantly lower FA values than B6xCAST mice (Figure 5C), suggesting lower axon coherence, density, or myelination in B6xD2 than B6xCAST mice. B6 mice had significantly higher MD and ISOVF than the progeny of all other crosses, again suggesting reduced cellular density and increased CSF content in this ROI in B6 mice. B6xCAST and B6xFVB mice had significantly higher FA values than the progeny of all other crosses, suggesting relatively high neurite coherence, axon myelination, and/or axon density in B6xCAST and B6xFVB mice. B6 mice were observed to have significantly lower OD than B6xD2 and B6xWSB mice. These differences in OD may result from a generally low concentration of cell bodies and their attached dendritic processes in B6, which is further supported by the significantly lower FA and significantly higher MD and ISOVF values in B6 than B6xCAST mice.

#### 3.3.4. Medial pallidum

In this ROI, B6xD2 mice had significantly higher MD values than B6xFVB mice (Figure 5D). Additionally, B6 mice had significantly higher MD values than the progeny of all other crosses and significantly higher ISOVF values than B6xCAST, B6xFVB, and B6xWSB mice. No significant differences in FA values were observed across strains. However, B6 mice had significantly higher OD values than B6xD2, B6xFVB, and B6xWSB mice. Therefore, differences in MD between B6 and B6xCAST mice can be explained by higher CSF content in the medial pallidum in B6 mice than B6xCAST mice; differences in MD between B6 and B6xD2 mice can be explained by greater neurite angular variation in B6 than B6xD2 mice; and differences in MD between B6, B6xFVB, and B6xWSB mice can be explained by both increased CSF volume and neurite angular variation in B6 medial pallidum. Significantly higher OD was observed in B6xCAST mice than B6xFVB mice, suggesting greater dendritic branching or higher dispersion of medial pallidum axons in B6xCAST mice than B6xFVB mice.

### 3.4. Multiple dMRI metrics suggested strain-driven differences in CSF content, cellular density, and neurite organization in two hindbrain gray matter ROIs

#### 3.4.1. Periventricular zone

**FIGURE 6:**
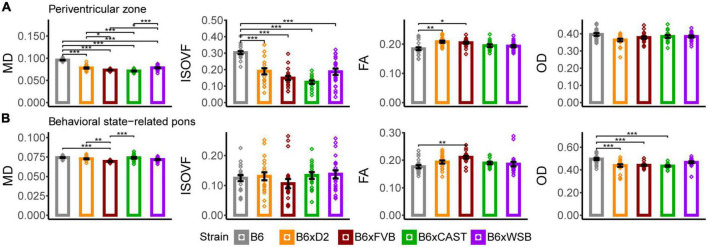
The periventricular zone and behavioral state-related pons were the hindbrain gray matter ROIs whose microstructure, as conveyed by dMRI, was most sensitive to strain. MD, ISOVF, FA, and OD strain differences in the periventricular zone **(A)** and behavioral state-related pons **(B)** suggested strain-dependent differences in hindbrain gray matter neurite organization, cell density, and CSF content in 3-month-old mouse brains. Bars represent data from 5XFAD and Ntg animals collapsed together and grouped by strain because no 5XFAD genotype effects were observed. Bar heights represent strain averages. Bar color corresponds to strain. Error bars represent standard error on the mean. Significance was determined by Tukey’s honest significance test following one-way ANOVAs testing for effects of strain on dMRI metrics (see Section “2 Materials and methods”), with resulting *p*-values FDR-adjusted (*p_*corrected*_ < 0.05; **p_*corrected*_ < 0.01; ***p_*corrected*_ < 0.001).

B6xD2 and B6xWSB mice had significantly higher MD values than B6xCAST and B6xFVB mice (Figure 6A), suggesting a lower concentration of cell bodies in B6xD2 and B6xWSB mice in the periventricular zone compared to B6xCAST and B6xFVB mice. Additionally, as observed in various white matter and forebrain gray matter ROIs, B6 mice had significantly higher MD and ISOVF values than the progeny of all other crosses in this cohort. Again, these results suggested higher CSF content in the B6 periventricular zone than those of the progeny of other crosses studied herein.

B6 mice also had significantly lower FA than B6xD2 and B6xFVB mice. Since the periventricular zone is a dense cluster of small cell bodies (Broadwell and Bleier, 1976) which receives projections from other brain regions (Bouret et al., 2004), these differences are likely attributable to higher incoming axon densities in the periventricular zones of B6xD2 and B6xFVB mice than B6 mice, consistent with the relatively low axon and cell densities implied by high MD and ISOVF values observed in many B6 ROIs.

#### 3.4.2. Behavioral state-related pons

B6xFVB mice had significantly lower behavioral state-related pons MD than B6, B6xD2, and B6xCAST mice, and no significant ISOVF differences were observed across strains (Figure 6B). Additionally, B6 mice had significantly lower FA than B6xFVB mice and significantly higher OD than B6xCAST, B6xD2, and B6xFVB mice. B6xWSB mice were observed to have significantly lower AxD values than B6xCAST mice (Supplementary Figure 2C), and B6xFVB mice had significantly lower AxD values than B6 and B6xCAST mice and significantly lower RD values than the progeny of all other crosses. Altogether, these results suggested (a) higher density of cell bodies and higher abundance, coherence, and/or myelination of incoming axonal projections in the behavioral state-related pons of B6xFVB mice than the progeny of other crosses in this cohort and (b) reduced axon content and/or greater dendritic complexity in B6 mice than the progeny of other crosses in this cohort.

### 3.5. 5XFAD genotype significantly influenced AxD in the stria medullaris

**FIGURE 7:**
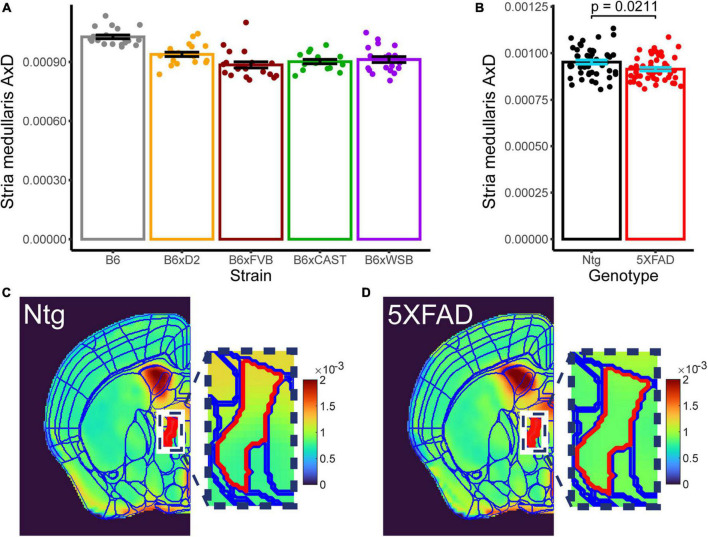
Effects of strain and 5XFAD genotype are observed in stria medullaris AxD. Strain [**(A)**, collapsed across genotypes] and 5XFAD genotype [**(B)**, collapsed across strains] differences in stria medullaris AxD values are shown. Individual points represent individual AxD values from each mouse, bar heights represent strain or 5XFAD genotype averages, and error bars represent standard error on the mean. Representative AxD images for **(C)** Ntg and **(D)** transgenic mice with a common scaling for AxD voxel values are shown. Borders for the Allen Brain Atlas are superimposed over the left hemisphere of each image. The stria medullaris is in red, and actual voxel values for the stria medullaris are depicted in the zoomed-in image.

Only one microanatomical measure showed a significant effect of 5XFAD genotype, which was of special interest in this study. AxD in the stria medullaris showed both a significant effect of strain (Figure 7A, *p* 3.066*10^–10^, three-way Type III ANOVA, FDR-adjusted) and 5XFAD genotype (Figure 7B, *p* 0.021, 3-way Type III ANOVA, FDR-adjusted; for representative images, see Figures 7C, D), but no strain*5XFAD genotype interaction. AxD values were higher in Ntg than transgenic mice, suggesting inflammation or another insult to axon health that impaired water diffusion along the length of stria medullaris axons in transgenic mice.

### 3.6. Strain exhibited the largest significant effects on baseline freezing and CFM performance, a measure of memory which formed correlations that were significant prior to FDR adjustment with dMRI measures in ROIs implicated in AD

**FIGURE 8:**
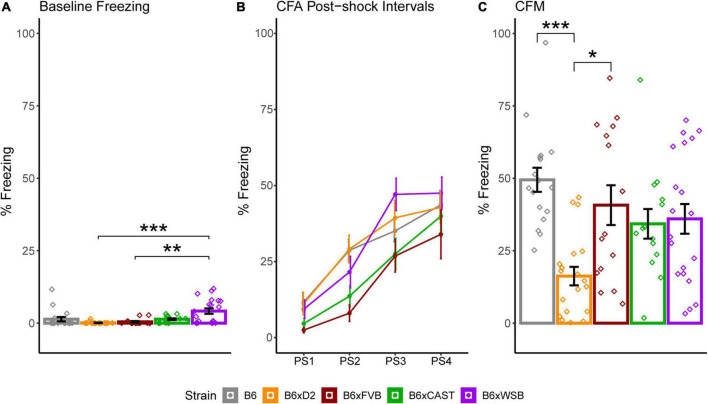
Strain differences in baseline freezing, contextual fear acquisition (CFA) percent freezing, and contextual fear memory (CFM) percent freezing conveyed minor differences in baseline activity and more robust strain differences in long-term memory acquisition. **(A)** Percent of time freezing during CFA baseline, shown to demonstrate innate differences in freezing among strains unrelated to the foot shock stimulus. CFA baseline freezing was significantly higher in B6xWSB mice than B6xD2 mice and B6xFVB mice (Tukey’s honest significance test, post 1-way Type I ANOVA, FDR-adjusted, *p_*corrected*_ < 0.05; **p_*corrected*_ 0.01; ***p_*corrected*_ < 0.001). **(B)** Strain differences in percent freezing for each post-shock CFA time bin conveyed similar short-term memory acquisition in all strains. **(C)** Strain differences in CFM percent freezing. CFM percent freezing was significantly higher in B6 and B6xFVB mice than B6xD2 mice (Tukey’s honest significance test, post 1-way Type I ANOVA, FDR-adjusted, *p_*corrected*_ < 0.05; **p_*corrected*_ < 0.01; ***p_*corrected*_ < 0.001). Effects of strain, sex, and 5XFAD genotype on CFA baseline freezing and CFA and CFM percent freezing were assessed with independent Type III, 3-way ANOVAs. Data from 5XFAD and Ntg animals were collapsed together to calculate strain means because no 5XFAD genotype effects were observed. Bar and line color correspond to different strains. Bar heights represent strain means. Error bars represent standard error on the mean. Individual points represent data from individual mice.

To determine how brain microanatomy related to cognitive health in our diverse mouse panel, the mice in this cohort underwent CFC (see Section “2 Materials and methods”) at 5 months of age. Baseline freezing, CFA PS4 percent freezing, and CFM percent freezing were calculated for each mouse. Effects of strain, sex, and 5XFAD genotype were assessed for baseline freezing, CFA PS4 percent freezing, and CFM percent freezing *via* Type III ANOVAs. Significant effects of strain (Figure 8A), sex, and strain*sex interaction (Supplementary Figure 4A) were observed on baseline freezing. No effects of strain, sex, 5XFAD genotype, or interactions were observed on CFA PS4 percent freezing (Figure 8), a measure of short-term memory. However, significant effects of strain (Figure 8), strain*sex interaction (Supplementary Figure 4B), and sex*5XFAD genotype interaction were observed on CFM percent freezing (Supplementary Figure 4C), a measure of long-term memory. Similarly to dMRI values, strain had the largest effect size out of all factors on baseline freezing and CFM percent freezing, as conveyed by ANOVA-derived F values and sums of squares (Table 3).

**FIGURE 9:**
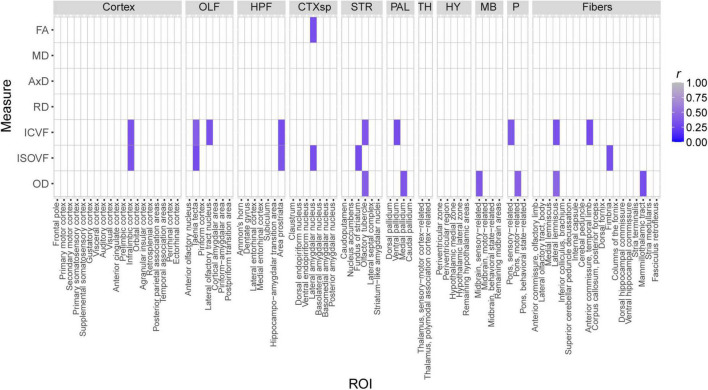
Nominally significant Pearson correlations (*r*) between dMRI measures and CFM percent freezing, a measure of fear memory strength. Tile color represents the strength of the correlation. All non-significant correlations are indicated by white tiles.

The effects of strain and strain*sex interactions on CFM increased our confidence in the idea that heritable biological factors may influence cognitive health by altering brain microstructure. Therefore, we calculated Pearson correlations between CFM percent freezing and each dMRI measure generated for this study. Correlations were only generated for Ntg mice, as 5XFAD mice have been previously demonstrated to exhibit robust changes in neural AD pathology between three and 6 months of age (Oblak et al., 2021). After correction for multiple comparisons, none of these correlations were significant. However, 22 correlations were significant prior to FDR adjustment (Figure 9).

## 4. Discussion

The brain reserve hypothesis stems from the observation that cognitive performance varies across individuals with similar levels of AD-related pathology (Stern, 2012). According to this idea, neural features that vary across individuals with different intrinsic biological characteristics may prevent cognitive decline despite onset of pathology. Therefore, studying the biological underpinnings of variation in neural features across mice with different genetic backgrounds may uncover sources of brain reserve. Recent evidence has suggested that brain microstructure varies with natural genetic variation, AD transgenic status, and sex (Parra et al., 2015; Sánchez-Valle et al., 2016; Harrison et al., 2020; Wang et al., 2020) and that brain microstructure is related to cognitive function (Scott et al., 2017; Mayo et al., 2019; Zavaliangos-Petropulu et al., 2019; Raghavan et al., 2021). In light of these findings, we performed DTI and NODDI imaging in mature adult mice at 3 months of age to identify ROI-specific variation in neural microarchitecture influenced by strain, sex, and the 5XFAD transgene. At 5 months of age, after preliminary analysis had demonstrated effects of strain on brain microstructure and after mice had recovered from anesthesia, these mice underwent CFC. As mice between three and 6 months of age exhibit similar health and macro- and microstructural neural features (Flurkey et al., 2007; Hammelrath et al., 2016; Kerkenberg et al., 2021), this experimental timeline enabled us to investigate (a) if brain microstructure varied with strain, sex, or 5XFAD genotype, (b) if short- and long-term fear memory varied with strain, sex, or 5XFAD genotype, and (c) if strain-related dMRI measurements could predict cognitive health in Ntg mice, thus determining the likelihood that brain microstructure confers brain reserve to AD.

### 4.1. Brain microstructure and memory were most sensitive to strain

Our dMRI results confirmed the sensitivity of brain microstructure to mouse strain. Strain had a significant effect on dMRI measures in most ROIs examined, and strain effects were far more frequent than effects of sex and strain*sex interactions, replicating previous findings (Wang et al., 2020). Additionally, the number of strain effects exceeded those of the 5XFAD transgene and strain*sex*5XFAD genotype interactions, and neither strain*5XFAD genotype nor sex*5XFAD genotype interactions were observed. While significant effects of strain were observed in most ROIs studied herein (Figure 2A), effect sizes of strain were largest for microanatomical features in the hippocampal formation, cortical subplate, hypothalamus, and fiber tracts (Figure 2B), all of which are memory- and AD-relevant brain areas (Hyman et al., 1984; Simić et al., 1997; Amorapanth et al., 2000; Giannakopoulos et al., 2003; Maren and Quirk, 2004; Bird and Burgess, 2008; Butler et al., 2018; Colon-Perez et al., 2019; Liao et al., 2021; Jordan et al., 2022; Tsui et al., 2022), indicating the potential of these microanatomical features to confer brain reserve against AD.

Additionally, to model how individual genomes in humans contribute to variability in brain microstructure, we calculated the heritability of strain means (*h*^2^_*rix*_) for each dMRI value in each ROI (see Section “2 Materials and methods”). Using multiple biological replicates of mice with the same genomes, as we did in this study, our study was better powered to understand the role of natural genetic variation in determining brain microstructure than any human study. Our *h*^2^_*rix*_ values demonstrated strong heritability of all dMRI measures in all ROIs (see Figure 3A). Therefore, it is evident that an individual’s genome, independent of causal AD mutations, influences brain microstructure early in life. A crucial extension of this finding is that there exist genetic drivers of brain microstructure that may serve as genetic modifiers of cognitive outcomes in AD, which holds promise for identifying novel therapeutic targets against AD-related cognitive decline. However, it should be noted that *h^2^_*rix*_* values do increase with the number of animals per strain/sex/5XFAD genotype group, so our *h^2^_*rix*_* values will be somewhat higher than the *h*^2^ heritability values like those reported in previous studies (Ashbrook et al., 2018, 2021).

The relevance of genetic background to brain microstructure was further probed by examining which ROIs had multiple dMRI measures with significant effects of strain. Excitingly, it was found that 10 ROIs had five or more dMRI measures with significant strain effects, and each of these 10 ROIs (cortical amygdalar area, lateral septal complex, dorsal hippocampal commissure, ventral hippocampal commissure, periventricular zone, lateral amygdalar nucleus, stria medullaris, fimbria, medial pallidum, and behavioral state-related pons) have previously been related to memory and AD (Amorapanth et al., 2000; Maren and Quirk, 2004; Castaño et al., 2013; Badea et al., 2016; Butler et al., 2018; Colon-Perez et al., 2019; Kim et al., 2019; Postans et al., 2020; Beardmore et al., 2021; Falangola et al., 2021; Liao et al., 2021; Noto et al., 2021; Jordan et al., 2022; Juárez-Leal et al., 2022; Tsui et al., 2022), again suggesting a relationship between brain microstructure and AD outcomes.

Of particular interest to this study were any observed effects of the 5XFAD transgene on DTI and NODDI measures. Previous characterization of B6-5XFAD transgenic mice have indicated the presence of amyloid beginning at 2 months of age (Giannoni et al., 2016), which has been suggested to increase RD and lower AxD and relative anisotropy, a measure similar to FA, in mouse white matter (Sun et al., 2014). Non-5XFAD animal models of amyloid pathology demonstrate early alterations in DTI measures at 2 months of age (Manno et al., 2019; Falangola et al., 2021). In white matter specifically, axon counts have been reported to be lower in amyloid-injected mice (Sun et al., 2014), and axonal dilatations containing amyloid-β have been observed in 3-month-old B6-5XFAD mice (Jawhar et al., 2012). However, no studies to date have assessed the effects of the 5XFAD transgene in genetically diverse mouse panels, which may explain the low prevalence of 5XFAD genotype effects observed in the set of dMRI measures described herein: only one ROI, the stria medullaris, had a significant effect of 5XFAD genotype. Additionally, amyloid pathology progresses with age in 5XFAD mice (Giannoni et al., 2016; Oblak et al., 2021), so more 5XFAD genotype-related alterations in dMRI measures may occur as mice age.

Mouse behavior was also influenced by strain. Strain exhibited the largest effects on baseline freezing and CFM percent freezing, as demonstrated by the large sums of squares and *F* values corresponding to strain (see Table 3). It follows that strain is the largest driver of baseline mouse activity in novel environments, which baseline freezing measures, and long-term memory, which CFM percent freezing measures, in 5-month-old mice. However, baseline freezing and CFM percent freezing are also sensitive to sex, as a significant main effect of sex and a significant strain*sex interaction were observed on baseline freezing and as significant strain*sex and sex*5XFAD genotype interactions were observed on CFM percent freezing. Between four and 6 months of age, 5XFAD mice on a C57BL/6J background begin to increase expression of amyloid substantially (Oblak et al., 2021). Therefore, sex-specific protective effects in response to the 5XFAD transgene may explain the observed improvement in CFM performance in male 5XFAD mice compared to male Ntg mice (Supplementary Figure 4C).

### 4.2. White matter, forebrain gray matter, and hindbrain gray matter dMRI signals revealed patterns in microstructural neural features that may track cognitive strength in genetically diverse mice

Multimodal dMRI imaging revealed many microstructural feature differences across strains in our cohort. Amidst these microstructural differences, two patterns of strain differences in dMRI signals were observed.

#### 4.2.1. High MD and ISOVF values in B6 mice suggested low cell and neurite density and high CSF content, which may confer reserve against AD

Firstly, seven of the 10 ROIs with five or more dMRI measures with effects of strain (lateral septal complex, ventral hippocampal commissure, periventricular zone, lateral amygdalar nucleus, stria medullaris, fimbria, and medial pallidum) had significantly higher MD and ISOVF in B6 mice than in the progeny of all other crosses. Additionally, another ROI, the cortical amygdalar area, exhibited significantly higher MD in B6 mice than the progeny of all other crosses, and the mean ISOVF of B6 mice in the cortical amygdalar area was noticeably higher than that of the progeny of all other crosses in this study, although these differences did not reach significance. Following common interpretations of MD and ISOVF (see Table 1), these results suggested higher CSF and lower cell/neurite density in these ROIs in B6 mice.

Given previous findings relating neuron density, MD, and ISOVF to cognitive abilities, one may expect B6 mice in our cohort to have poor memory performance. In mice, higher neuron density in the BLA is associated with improved CFM performance (Yang et al., 2008), and neurogenesis-driven decreases in MD track spatial learning improvements during a related active place avoidance paradigm (Zhou et al., 2021). Additionally, higher cortical neuron counts in mice have been associated with better perceptual discrimination (Fang and Yuste, 2017). In humans, memory and cognitive health have been associated with lower MD (Scott et al., 2017; Mayo et al., 2019; Zavaliangos-Petropulu et al., 2019) and ISOVF (Radhakrishnan et al., 2020; Raghavan et al., 2021). Lastly, animals with similar absolute numbers of forebrain neurons have similar cognitive abilities even though their forebrain sizes differ (Olkowicz et al., 2016). Our results contrast these findings. Significant effects of strain and strain*sex interaction on CFM percent freezing were observed in our mouse cohort. B6 mice had the highest CFM percent freezing strain average, suggesting high memory performance, followed by B6xFVB, B6xWSB, B6xCAST, and B6xD2 in descending order. It should be noted that B6 CFM performance only differed significantly from that of B6xD2 mice, but the fact that supposed indicators of memory deficits (high ISOVF and MD) were exhibited in mice with strong memory performance is still noteworthy. Differences in baseline freezing were also observed among strains, with B6xWSB mice exhibiting significantly higher baseline freezing than B6xD2 and B6xFVB mice. Such differences in baseline freezing suggested that B6xWSB mice are less hyperactive or have a weaker inclination to explore novel environments than the other mice in this cohort. These differences between B6xWSB, B6xD2, and B6xFVB were less than 3% and unlikely to confound strain differences in CFA PS4 and CFM percent freezing.

There are several potential explanations for our results. Firstly, previous studies have not examined the relationships between memory/cognition and MD, ISOVF, or cell density in the ROIs examined in this study. One study examining neuron density across larger brain areas did not find significant relationships between memory tasks and neuron density in any brain region examined, including the hippocampus, anterior cerebral cortex, posterior cerebral cortex, and total cerebral cortex. Interestingly, this study does report negative correlations between neuron count in posterior and total cerebral cortex and performance on a spatial learning task that approach significance (Pearson’s coefficient ≤ −0.29, *p* ≤ 0.1) (Neves et al., 2020).

In addition, differences in MD and ISOVF may reflect glial differences between strains that may affect cognitive health. For example, healthy microglial activity may increase water diffusivity in the brain, as microglia prune synapses (Neumann et al., 2009), assist in neural apoptosis (Wakselman et al., 2008), and clear cellular debris (Schafer et al., 2012). These processes have been noted to improve circuit efficiency. Additionally, deficiencies in synaptic pruning during development have been shown to lead to increased local neural connectivity (as may be observed in ROI-based analyses like our own), which has been implicated in autism spectrum disorders (Belmonte et al., 2004). By promoting healthy neural circuit development, microglia may increase the proportion of “efficient” synapses in the brain; thus, more synapses will need to be lost in AD for cognitive symptoms to occur.

Furthermore, if developmental microglial activity is heightened in B6 mice, these mice may also exhibit robust microglial activity later in life. Microglia have been implicated in amyloid-β clearance in early stages of AD (Hickman et al., 2008), synapse formation, and synaptic recovery from damage (Brucato and Benjamin, 2020). Each of these functions may promote long-term neural and cognitive health in individuals with genetic predispositions to AD.

Lastly, CSF itself plays active roles in clearing toxins, including amyloid-β, from the brain and delivers critical nutrients to neurons *via* the glymphatic system (Jessen et al., 2015). Therefore, increased CSF content may nourish and protect neurons and synaptic circuits, complementing improved microglial activity in B6 mice to promote long-term cognitive health.

#### 4.2.2. B6xFVB mice exhibited high axon coherence in white matter tracts implicated in AD

B6xFVB mice exhibited higher FA than B6, B6xD2, and B6xCAST mice in the stria medullaris and the progeny of all other crosses in the fimbria and dorsal hippocampal commissure (Figures 4A, B, D). Importantly, each of these white matter tracts are shown to be involved in AD-related circuits or pathology (Miller et al., 1994; Hangya et al., 2009; Palop and Mucke, 2009; Colon-Perez et al., 2019; Kim et al., 2019; Falangola et al., 2021; Juárez-Leal et al., 2022; Tsui et al., 2022). Cumulatively, these results indicated that B6xFVB axon coherence is uniquely high in these AD-related white matter tracts. Given the relationship between white matter FA and cognitive health in mice and humans (Mayo et al., 2019; Wen et al., 2019; Fu et al., 2020), white matter coherence in the stria medullaris, dorsal hippocampal commissure, and fimbria may be a source of brain reserve. It is difficult to determine if this notion aligns with our study’s behavioral data. B6xFVB mice had the second highest CFM percent freezing strain average (Figure 8), suggesting high cognitive ability. However, differences in behavior across B6xFVB males and females are notable (Supplementary Figures 4A, B) and do not reflect the lack of sex differences in the stria medullaris (two-sample *t*-test, *p* = 0.240), fimbria (two-sample *t*-test, *p* = 0.765), or dorsal hippocampal commissure FA (two-sample *t*-test, *p* = 0.423) in B6xFVB mice (Supplementary Figure 5). Additionally, FA in the fimbria was significantly lower in B6 mice, which had the highest mean CFM percent freezing in our mouse cohort, than B6xFVB mice. Therefore, we tentatively conclude that axon coherence in the stria medullaris, fimbria, and dorsal hippocampal commissure may confer brain reserve against AD but acknowledge that other neurological features are likely to mitigate the effects of axon coherence in these white matter tracts on cognitive decline.

### 4.3. The stria medullaris may be an early site of pathology in genetically diverse mice

The stria medullaris is the only ROI in this study shown to have a significant effect of 5XFAD genotype. Stria medullaris AxD values were significantly greater in Ntg than transgenic 3-month-old mice, suggesting that the 5XFAD transgene negatively impacts stria medullaris axon coherence, density, or general structural integrity. The stria medullaris projects from the septum, hypothalamus, and pallidum, all of which are implicated in AD pathology, to the habenular nuclei (Juárez-Leal et al., 2022), all of which are implicated in AD pathology. The hypothalamus controls feeding and metabolic activity (Saper and Lowell, 2014), and its atrophy and dysfunction occur early in AD development (Ishii and Iadecola, 2015). Weight loss commonly occurs in prodromal and clinical stages of AD (White et al., 1996; Sergi et al., 2013), and mouse models of AD exhibit hypothalamic gene expression levels that correlate to both weight loss and cognitive deficits (Dai et al., 2022). The septum and pallidum have been reported to undergo AD-related morphological changes (Teipel et al., 2005; Gan et al., 2017), elicit hippocampal theta oscillations (Hangya et al., 2009; Chee et al., 2015), and modulate hippocampal epileptogenesis (Miller et al., 1994), which is observed in human AD patients (Lam et al., 2017) and AD mouse models (Palop and Mucke, 2009). Lastly, the habenula is implicated in motivation and behavioral regulation (Hikosaka et al., 2008). Therefore, impaired signaling between the habenula and the hypothalamus, two motivation-related regions, caused by stria medullaris pathology may explain the apathy commonly observed in AD patients (Landes et al., 2001; Teixeira and Caramelli, 2006; Gitlin et al., 2012). It is also worth noting that microstructure of the hypothalamus, septal nuclei, and medial pallidum is heavily influenced by strain, as our results indicate. Ultimately, genetically influenced microstructural features in the hypothalamus, septum, and pallidum may interact with early pathology in the stria medullaris to alter susceptibility to AD (Oblak et al., 2021).

### 4.4. Limitations

Our ability to resolve anatomical brain features and thus align them to the Allen Brain Atlas with feature-based registration was limited by our image resolution. Our voxel size for DTI and NODDI images was 164 × 164 × 750 μm. At this resolution, brain region borders may not be clear, especially for small nuclei like those found in the thalamus and hypothalamus, which may lead to partial volume averaging and other confounds of results. Upsampling of our images through the *flirt* function in FSL mitigated this issue, but feature alignment was still less reliable for some small nuclei and white matter tracts. To address this issue, we collapsed small nuclei belonging to shared substructures of the brain (for example, collapsing CA1, CA2, and CA3, which collectively make up Ammon’s horn) if those nuclei have smaller dimensions than those of our raw image voxels. These dimensions were estimated using the Allen Interactive Atlas Viewer.^6^

Our large voxel thickness (750 μm) also created border effects. As shown in Supplementary Figures 1B, C, border effects limited our ability to resolve outer cortical layers and ventral nuclei of the hypothalamus. We mitigated these issues by averaging values from all cortical layers in each cortical region and by omitting the medial eminence, a small nucleus at the ventral border of the hypothalamus, from this study.

Regarding NODDI, our ability to fit NODDI parameters (ICVF, ISOVF, and OD) with high confidence was dependent on the strength of our gradient coil and number of gradient directions. Typically, NODDI imaging utilizes a large b value of at least 2000 sec/mm^2^ (Zhang et al., 2012; Colgan et al., 2016; Crombe et al., 2018; Colon-Perez et al., 2019; Zamani et al., 2022). However, hardware limitations limited our maximum b value to 1200 sec/mm^2^. Additionally, to accommodate the multimodal imaging this study involved (see Section “2 Materials and methods”), we limited the number of gradient directions to 6 for *b* = 600 sec/mm^2^ and 46 for *b* = 1200 sec/mm^2^. Early work investigating the NODDI model has indicated that protocols similar to the one used herein generate NODDI images comparable to a highly accurate, “ground truth” 4-shell protocol (Zhang et al., 2012). The reliability of our protocol is supported further by two studies which report ISOVF and OD values within range of those reported in Zhang et al. (2012): Cruz-Almeida et al. (2021), who used a small b value of 100 sec/mm^2^ and large b value of 1000 sec/mm^2^ for NODDI imaging; and by Colon-Perez et al. who obtained 6 gradient directions for a small b value of 600 sec/mm^2^ and 46 gradient directions for a large b value of 2000 sec/mm^2^ for NODDI imaging. The ICVF values generated by Cruz-Almeida et al. (2021) are slightly outside the range reported in Zhang et al. (2012) but close enough to this range to be considered to reliably reflect underlying tissue differences between groups. Some of our images (see Figures 3B–F) also demonstrated higher white matter ISOVF than gray matter ISOVF, a pattern consistent with previous NODDI studies using conventional NODDI image acquisition parameters (Colgan et al., 2016; McCunn et al., 2019). Additionally, our NODDI values were within range of those reported in NODDI studies utilizing b values of 2000 or higher [for example, see Colon-Perez et al. (2019)], and our SNR for diffusion images (see Section “2 Materials and methods) are comparable to images generated with higher field strengths (Colon-Perez et al., 2019). Lastly, and most importantly, our protocol yielded many DTI and NODDI values that exhibited many strain effects and high heritability, replicating previous findings on the relationship between brain microstructure and natural genetic variation (Wang et al., 2020). Such results cannot be explained by spurious technical variation; in other words, our protocol is sensitive enough to detect genetically related differences in mouse brain microstructure, the primary objective of this study. Ultimately, as MD and ISOVF are independent measures that yield complementary but differing information regarding brain microstructure, ISOVF enhanced our analysis of brain microstructure and allowed us to determine the contribution of CSF to MD signals. Nonetheless, we acknowledge that technical variation may have been introduced into our NODDI images because of our low b value, especially for ICVF images.

A potential concern with our CFC protocol is that a 2-month time gap between MRI imaging and fear conditioning may confound the relationships we observed between dMRI measures and CFC. As this study’s primary goal was to assess variation in brain microanatomy across strain, sex, and 5XFAD genotype in our genetically diverse AD mouse cohort, we only considered testing cognitive health in mice after observing strain-related variation in many dMRI measures. Performing CFC in mice at 5 months of age allowed mice to recover from anesthesia and the stress of MRI imaging and enabled us to prepare for and arrange CFC assays. We are confident that our experimental design did not confound the correlations between dMRI values and CFM observed in Ntg mice (Figure 9) because Ntg mice do not exhibit behavioral differences between 3 and 6 months of age (data not shown), and because mice are not expected to show differences in microstructure between 3 and 5 months of age (Flurkey et al., 2007; Hammelrath et al., 2016; Kerkenberg et al., 2021). It is important to mention that no correlations between dMRI values and CFM performance were significant after FDR-adjustment. Therefore, we interpret correlational values displayed in Figure 9 as potential relationships between brain microstructure and memory that may become significant as memory and structural integrity decline with age (Neuner et al., 2019; Onos et al., 2019).

Lastly, our scanning protocol failed to capture the entirety of the medulla, cerebellum, and olfactory bulb (Supplementary Figure 1C). Thus, data from ROIs in the cerebellum and medulla were removed from our analysis. Regarding the olfactory bulb, the main and accessory olfactory bulbs were omitted due to incomplete coverage. The anterior olfactory nucleus was also not fully covered. However, because this nucleus extends far into the regions of the brain that were covered, and only a small part of the anterior olfactory nucleus was not covered in each image (determined through visual inspection), the anterior olfactory nucleus was included in this study.

Ultimately, due to the technical limitations of our study described herein, we believe that negative results presented in this study may not reflect a lack of genetic effects on the microstructure of some ROIs, while positive results reflect strong genetic effects on the microstructure of other ROIs.

## 5. Conclusion

Our study supports previous findings reflecting the influence of natural genetic variation on brain microanatomy of mature adult mice. By filtering brain regions by the sensitivity of their microanatomy to strain, we identified several ROIs implicated in AD, suggesting that brain microanatomy may be a mechanism by which genetic variation confers brain reserve to AD. It is worth noting that no effects on ICVF, a measure specifically analyzing the volume fraction of neurites, were observed in this study. Therefore, any inferred differences in neurite density may be subtle, or variations in MD, typically linked to changes in cell and neurite density, may reflect differential densities of glial cells or lower densities of neural somas accompanied by increased levels of dendritic branching. Alternatively, because our image acquisition protocol used a relatively low b value for NODDI imaging, a lack of ICVF effects may be due to increased technical variation in ICVF images, which have been demonstrated to be especially sensitive to b values. We also are the first to characterize memory strength in our genetically diverse mouse panel, as our mice underwent CFC as mature adults. A significant strain*sex interaction was observed on baseline freezing, suggesting that strain interacts with sex to alter long-term memory and hyperactivity or novel space exploration. A sex*5XFAD genotype interaction was observed for CFM percent freezing, indicating that sex may mitigate the impact of the 5XFAD transgene on long-term memory. Our experiment timeline allowed us to directly relate cognitive ability to brain microstructure in Ntg mice after determining that brain microstructure varied with strain. While no observed correlations between behavior and microstructure-related dMRI measures were significant after FDR-adjustment, we observed 22 correlations that were significant prior to FDR-adjustment. Additionally, B6 mice, the mice in our panel with the highest memory performance, tended to have high MD and ISOVF in AD-relevant ROIs. The combination of MD and ISOVF classically indicates high CSF volume and low neuron density, which has traditionally been related to lower cognitive function. Additionally, B6xFVB mice, another strain with high CFM performance, exhibited high white matter FA, suggesting that white matter coherence may contribute to memory health in mice. Future work will assess further how cell densities in this mouse panel relate to cognitive outcomes.

## Data availability statement

The original contributions presented in this study are included in the article/Supplementary material, further inquiries can be directed to the corresponding author.

## Ethics statement

The animal study was reviewed and approved by the Institutional Animal Care and Use Committee at The Jackson Laboratory. Animal health was monitored routinely throughout the duration of this study.

## Author contributions

TM conducted quality control inspections on all images, performed statistical analyses on behavioral and processed MRI data, and wrote the manuscript. AD provided code for early statistical analyses and the generation of some figures. SS generated panels for some figures herein. MT assisted in initial data procurement. MT, MF, AD, SS, JW, and SZ provided feedback on manuscript drafts. SZ provided other authors much guidance on project methodology and performed image acquisition, preprocessing, and visual quality control inspections. MF provided crucial intellectual support in data interpretation. CK, MF, and IK built the initial conceptual framework for the project. CK, AD, SS, SZ, MF, and IK provided key insights and mentorship throughout data acquisition and analysis. All authors contributed to the article and approved the submitted version.

## Footnotes

1
https://osf.io/t9vqn/

2
https://github.com/SebastianoF/bruker2nifti

3
http://dsi-studio.labsolver.org

4
https://fsl.fmrib.ox.ac.uk/fsl/fslwiki/

5
https://github.com/daducci/AMICO

6
http://atlas.brain-map.org/atlas?atlas=1#atlas=1&plate=100960224&structure=549&x=5280.099051339286&y=3744.082903180804&zoom=-3&resolution=NaN&z=6
