# Supplementary material for: Leveraging genetic diversity in mice to inform individual differences in brain microstructure and memory
Source: Front Behav Neurosci. 2023 Jan 10;16:1033975. doi: 10.3389/fnbeh.2022.1033975 (PMC9871587; doi:10.3389/fnbeh.2022.1033975)
Supplement: Supplementary file 1 [file Presentation_1.pdf]

Supplementary Material

Leveraging Genetic Diversity in Mice to Inform Individual Differences in Brain Microstructure and Memory

Thomas J. Murdy[1], Amy R. Dunn[1], Surjeet Singh[1], Maria A. Telpoukhovskaia[1], Shanrong Zhang[1], Jacqueline K. White[1], Itamar Kahn[2], Marcelo Febo[3], Catherine C. Kaczorowski[1*]

[1]The Jackson Laboratory, Bar Harbor, ME, United States

[2]Department of Neuroscience and Zuckerman Mind Brain Behavior Institute, Columbia University, NY, United States

[3]Department of Neuroscience, University of Florida College of Medicine, Gainesville, FL, United States

*** Correspondence:** Catherine C. Kaczorowski, catherine.kaczorowski@jax.org

1 Supplementary Figures

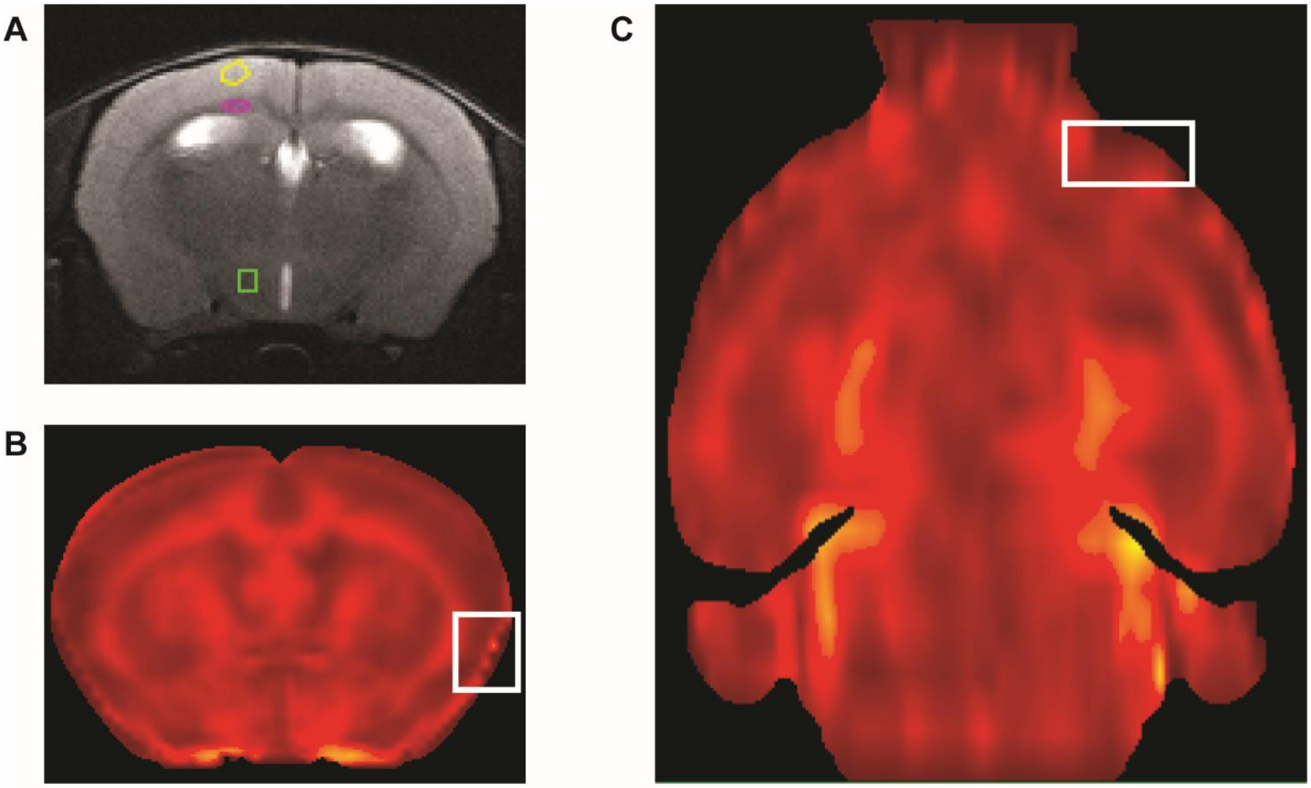

Supplementary Figure 1. Representations of image limitations and regions selected for SNR calculations. A) The regions utilized to calculate SNR for all image types (T_2, dMRI b = 0 sec/mm2, and dMRI b = 1200 sec/mm2). The region outlined in yellow was used to calculate the SNR for dorsal regions, while the region outlined in green was used to calculate the SNR for ventral regions.

B, C) Border effects and incomplete coverage of forebrain and hindbrain ROIs are exhibited in dMRI images. Representative images of border effects, highlighted in white boxes **(B, C)**, and limits in coverage of olfactory bulb, medulla, and cerebellum **(C)** in dMRI images are shown.

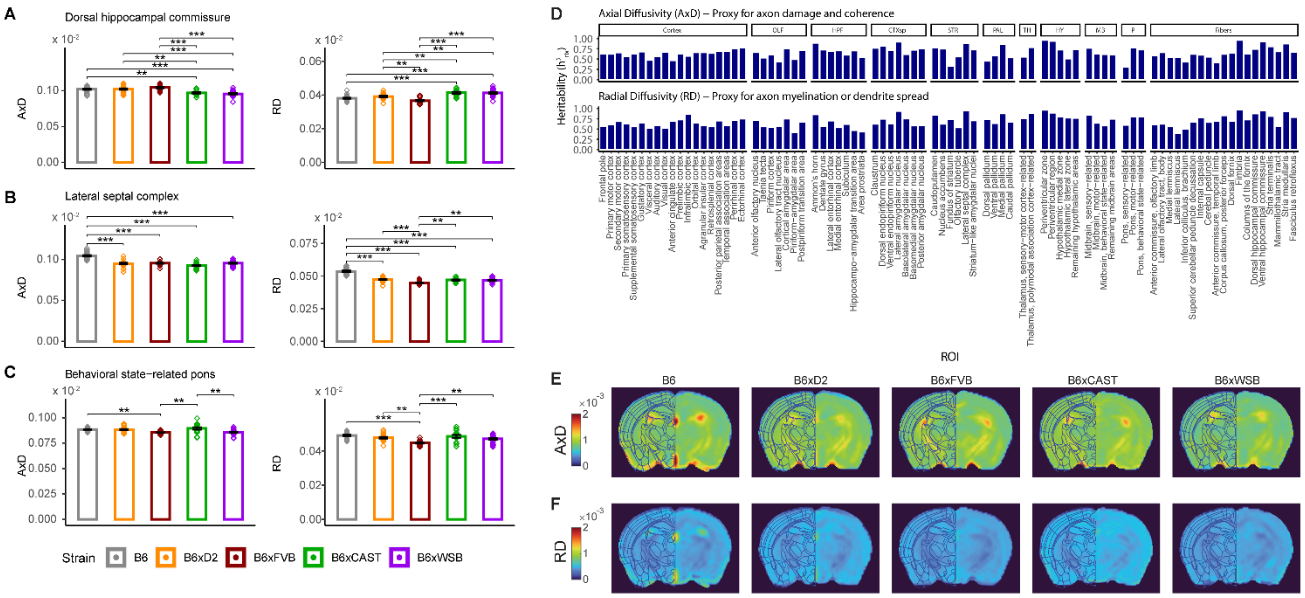

Supplementary Figure 2. AxD and RD values showed robust strain differences and heritability and exhibited differing patterns of strain differences in the dorsal hippocampal commissure, lateral septal complex, and behavioral state-related pons. A-C) Strain differences in AxD and RD in the **(A)** dorsal hippocampal commissure, **(B)** lateral septal complex, and **(C)** behavioral state-related pons. **D)** Group size-adjusted heritabilities (h^2_{rix}) of AxD and RD demonstrated strong influences of natural genetic variation (strain) on AxD and RD in most ROIs. **E, F)** Representative images for **(E)** AxD and **(F)** RD.

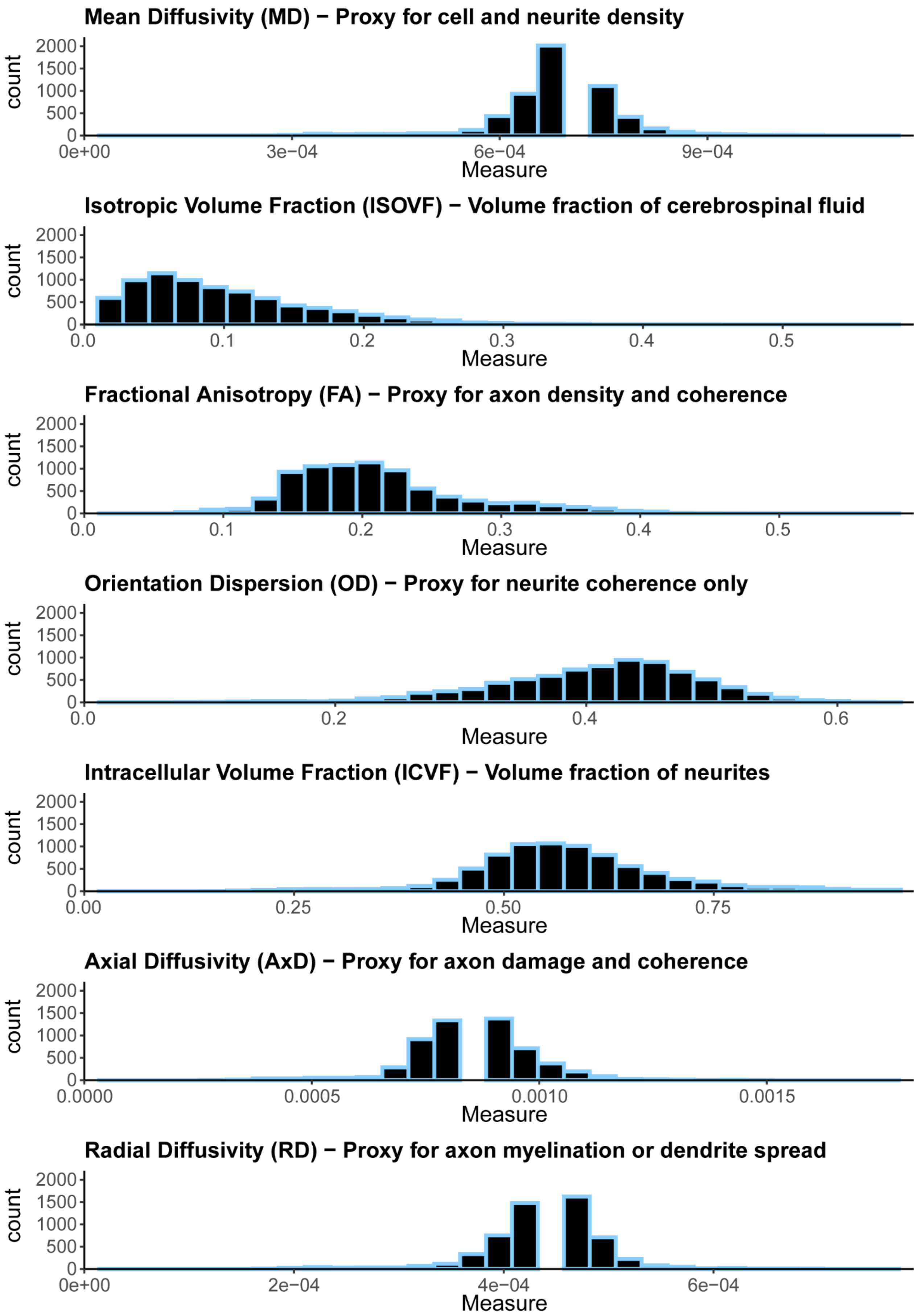

Mean Diffusivity (MD) − Proxy for cell and neurite density
count
0e+00
3e−04
6e−04
9e−04
Measure
Isotropic Volume Fraction (ISOVF) − Volume fraction of cerebrospinal fluid
count
Measure
Fractional Anisotropy (FA) − Proxy for axon density and coherence
count
Measure
Orientation Dispersion (OD) − Proxy for neurite coherence only
count
Measure
Intracellular Volume Fraction (ICVF) − Volume fraction of neurites
count
Measure
Axial Diffusivity (AxD) − Proxy for axon damage and coherence
count
0.0000
0.0005
0.0010
0.0015
Measure
Radial Diffusivity (RD) − Proxy for axon myelination or dendrite spread
count
0e+00
2e−04
4e−04
6e−04
Measure

Supplementary Figure 3. Distributions of dMRI measures obtained from all 83 ROIs examined in this study.

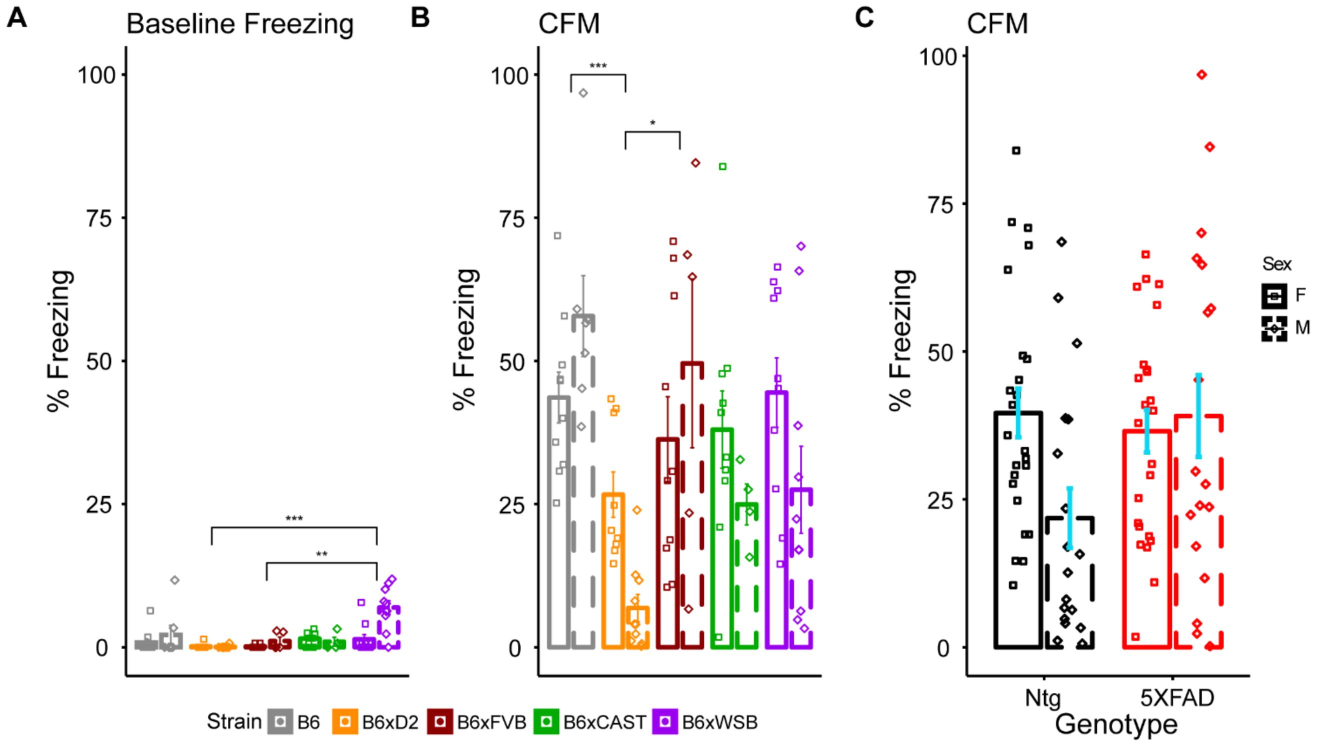

Supplementary Figure 4. Sex interacts with strain and 5XFAD genotype to influence baseline freezing and CFM percent freezing. A) Strain/sex group differences in baseline freezing representing the strain*sex interaction exhibited in baseline freezing. **B)** Strain/sex group differences in long-term memory, as conveyed by CFM, representing the strain*sex interaction exhibited in CFM percent freezing. **C)** Sex/5XFAD genotype group differences in CFM representative of the sex*5XFAD genotype interaction exhibited in CFM percent freezing. Results from 5XFAD and Ntg animals are collapsed together because no 5XFAD genotype effects on CFM were observed. Significance bars represent strain differences determined by Tukey's honest significance tests (post 1-way Type I ANOVA, FDR-adjusted, *: $p < 0.05$, **: $p < 0.01$, ***: $p < 0.001$, see Methods). Bar heights represent group means. Error bars represent standard error on the mean. Individual points represent data from individual mice.

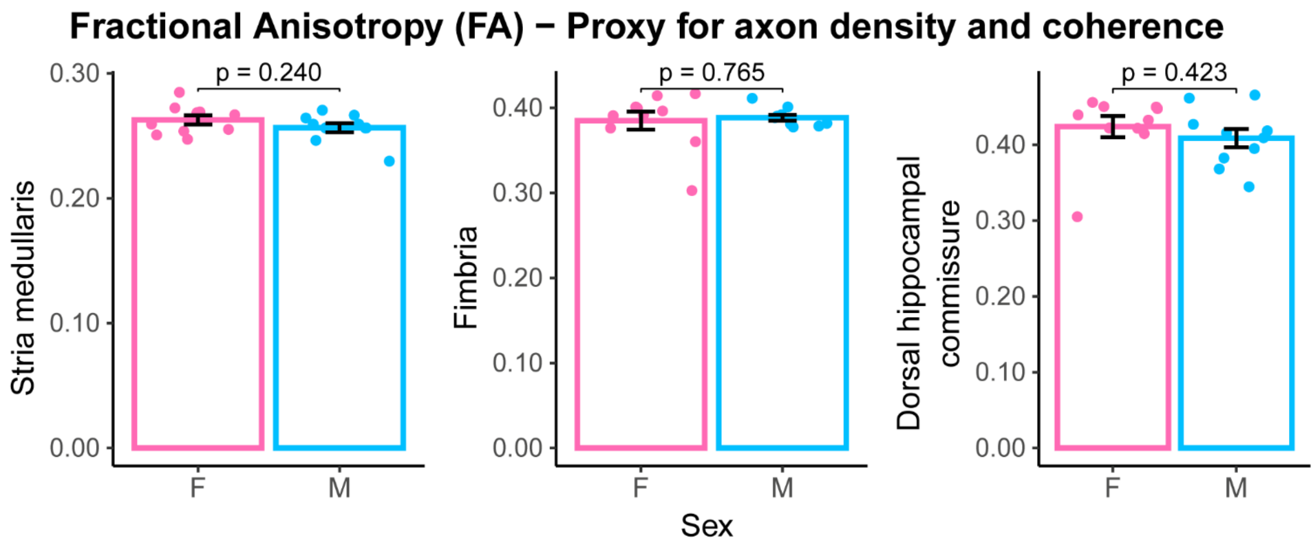

Supplementary Figure 5. No significant sex differences were observed in the FA of the stria medullaris, fimbria, and dorsal hippocampal commissure in B6xFVB mice. Bars represent data from 5XFAD and Ntg animals collapsed together and grouped by sex because no 5XFAD genotype effects were observed. Bar heights represent sex means, and error bars represent standard error on the mean. Significance was determined by two-way t-tests. p-values are presented above the bars in each bar plot.
